# Electropositive Magnetic Fluorescent Nanoprobe‐Mediated Immunochromatographic Assay for the Ultrasensitive and Simultaneous Detection of Bacteria

**DOI:** 10.1002/advs.202412421

**Published:** 2025-01-13

**Authors:** Jiaxuan Li, Zhengkang Li, Bingjie Wang, Qing Yu, Ting Wu, Chongwen Wang, Bing Gu

**Affiliations:** ^1^ Department of Clinical Laboratory Medicine Guangdong Provincial People's Hospital (Guangdong Academy of Medical Sciences) Southern Medical University Guangzhou Guangdong 510000 China; ^2^ School of Medicine South China University of Technology Guangzhou 510006 China

**Keywords:** antibody‐free electropositive probe, bacteria, electrostatic interaction, immunochromatographic assay, multiplex detection

## Abstract

Immunochromatographic assays (ICAs) provide simple and rapid strategies for bacterial diagnosis but still suffer from the problems of low sensitivity and high dependency on paired antibodies. Herein, the broad‐spectrum capture and detection capability of the antibody‐free electropositive nanoprobe are clarified for bacteria for the first time and an ultrasensitive fluorescent ICA platform is constructed for the simultaneous diagnosis of multiple pathogens. A magnetic multilayer quantum dot nanocomposite with an amino‐embedded SiO_2_ shell (MagMQD@Si^+^) is designed to enrich bacteria from solutions effectively, offer high luminescence, and reduce background signals on test strips, thus greatly improving the sensitivity and stability of ICA technique for pathogen. The superior performance of the MagMQD@Si^+^‐based ICA through the multiplex detection of three common pathogens is demonstrated, namely, *Pseudomonas aeruginosa*, *Streptococcus pneumoniae*, and *Salmonella typhimurium*, showing that this ICA possesses high sensitivity (8–40 cells mL^−1^), good reproducibility (relative standard deviation *<*5.4%), and high specificity for the three target bacteria. The clinical utility of the proposed method is verified through the detection of 30 real sputum samples from patients with bacterial respiratory infections, revealing that the MagMQD@Si^+^‐based ICA has massive potential as a powerful inspection tool for the rapid, sensitive, and ultrasensitive diagnosis of bacterial infections. [Correction added on 29 January 2025, after first online publication: *Streptococcus typhi* was corrected to *Salmonella typhimurium*.]

## Graphical abstract

An ultrasensitive fluorescent immunochromatographic assay (ICA) platform is reported for the first time for bacterial diagnosis with universal and multiplex detection ability based on the broad‐spectrum capture ability of electropositive MagMQD@Si^+^ for negatively charged bacterial cells.

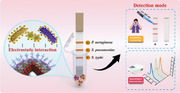

## 1 Introduction

In the long history of humanity, pathogenic bacteria have always been a major threat to human life and health.^[^ ^1^ ^]^ Bacterial infections are closely associated with numerous serious human diseases, such as pneumonia, infectious diarrhea, meningitis, sepsis, and urinary tract infections, which all result in hundreds of millions of hospitalizations around the world and over 7.7 million deaths each year.^[^ ^2^ ^]^ The timely identification of pathogens guides clinicians to perform accurate treatment and rational drug use and is therefore the most effective means to save lives. However, current mature detection technologies, such as bacterial culture, polymerase chain reaction (PCR), genomic sequencing, and mass spectrometry, still face shortcomings, including complex operations; long detection times (3–24 h); and requirements for clean lab spaces, professional technical staff, and expensive instruments (cost of PCR instruments >$6000; cost of sequenators >$100 000, and cost of mass spectrometers >$80 000).^[^ ^3^ ^]^ Therefore, developing a technology suitable for the rapid and on‐site screening of pathogens is urgently needed to meet the clinical needs of early diagnosis and treatment.

Immunochromatographic assays (ICAs) have become one of the most popular point‐of‐care testing (POCT) techniques because they integrate chromatographic separation and specific antibody–antigen recognition on a simple test strip, allowing the rapid, simple, and low‐cost detection of various biochemical targets (e.g., proteins, nucleic acids, and viruses) and do not rely on large instruments.^[^ ^4^ ^]^ Although many ICA techniques for bacteria based on detection reagents have been reported, these methods suffer from low sensitivity and detection throughput and poor versatility due to two aspects: First, given that bacteria have large sizes (0.5–4 µm), constructed ICA systems generally adopt a double‐antibody sandwich strategy that typically requires one antibody to label detection nanoprobes for bacterial binding and one antibody to modify the detection line of a test strip for capturing formed immunocomplexes.^[^ ^5^ ^]^ However, the preparation and selection of high‐performance antibody pairs for ICAs are time‐consuming, laborious, and costly.^[^ ^6^ ^]^ In addition, antibody molecules on the surfaces of nanoprobes are prone to interference from complex samples (e.g., high ion concentrations and harsh pH environments).^[^ ^7^ ^]^ Such interference reduces their biological activities and storage stability and finally decreases the stability and sensitivity of detection methods. Second, the performance of current nanoprobes is insufficient for bacteria detection. Traditional colorimetric nanoparticles (NPs), such as AuNPs and latex beads, exhibit weak sensitivities (10^4^–10^3^ cells mL^−1^ level).^[^ ^8^ ^]^ Newly developed signaling materials, such as Raman tags, fluorescence probes, and catalytic NPs, can effectively increase the sensitivity of ICAs for bacterial detection but still face the problems of poor stability in complex clinical samples and require antibody modification.^[^ ^9^ ^]^ In theory, exploiting a high‐performance antibody‐free nanoprobe with the features of enhanced signals, high stability and affinity for bacteria, and broad‐spectrum binding ability may solve the challenges encountered in the sensitive and accurate detection of pathogens on ICA platforms.

In recent years, many efforts have been made to develop antibody‐free biosensors for bacterial detection, and research results have proven that antibody‐free nanoprobe‐based ICA systems are applicable for rapid identification of pathogen.^[^ ^10^ ^]^ For example, Deng et al. reported that 2D MnO_2_ has good bacterial adsorption capacity and strong colorimetric signals and is suitable as the antibody‐free probe of an ICA for the diagnosis of *Salmonella enteritidis*.^[^ ^11^ ^]^ Zhang et al. showed that the Fe_3_O_4_@CuS nanostructure possesses strong capture ability for bacteria and successfully constructed a colorimetric and photothermal‐mode ICA for the detection of *Escherichia coli* O157:H7.^[^ ^12^ ^]^ The application of the antibody‐free probe reduced the dependence of the ICA technique on paired antibodies and eliminated antibody modification processes, effectively simplifying ICA preparation. However, current antibody‐free strategies still have the following major scientific problems that need solution: i) The mechanism of the binding of current antibody‐free probes to bacteria is unclear, and the broad‐spectrum detection ability of these probes has not been demonstrated. ii) Existing antibody‐free probe‐based ICAs can only detect one target pathogen, and their detection throughputs are insufficient for the rapid screening of multiple bacteria. iii) The detection limits of existing antibody‐free probe‐based ICAs for bacteria are generally higher than 10^2^ cells mL^−1^, which cannot meet the actual needs of clinical diagnosis.

Some previous studies have demonstrated that most bacteria (Gram‐positive and Gram‐negative bacteria) are electronegative in solutions over a wide pH range (4–10) because their cells possess net negative charges that are conferred by ionized phosphoryl and carboxylate substituents on the macromolecules of the outer cell envelope.^[^ ^13^ ^]^ On the basis of this characteristic, in this study, we propose a universal ICA detection platform for the ultrasensitive and simultaneous detection of multiple bacteria by using the well‐designed electropositive probe MagMQD@Si^+^. The multifunctional MagMQD@Si^+^ probe possesses a 200 nm Fe_3_O_4_ core to provide strong magnetic enrichment capacity, a multilayer inner shell formed by quantum dots (QDs) to generate superior luminous ability, and an amino‐modified SiO_2_ shell to provide the ability for the broad‐spectrum binding of bacteria and high liquidity for ICA detection. We reveal that the positively charged SiO_2_ shell of MagMQD@Si^+^ promotes strong electrostatic interactions with negatively charged pathogens, allowing the electropositive probe to capture and enrich multiple bacteria rapidly from complex solutions and enabling their ultrasensitive and highly specific detection on the antibody‐modified ICA strip. We demonstrate the universality and multiplex detection ability of our proposed method for bacteria by detecting three important bacterial pathogens, namely, *Pseudomonas aeruginosa*, *Streptococcus pneumoniae*, and *Salmonella typhimurium*. We selected these bacteria because they represent common respiratory, nosocomial infectious, and foodborne bacteria, respectively. Among these bacteria, *S. pneumoniae* is responsible for a substantial number of lower respiratory tract infections and fatalities among newborns and young children.^[^ ^14^ ^]^ *S. typhi* is closely linked to deaths in children aged 5–14 years, resulting in an estimated 49 000 fatalities.^[^ ^15^ ^]^ *P. aeruginosa* usually induces lower respiratory tract, bloodstream, and intra‐abdominal infections, causing more than half a million deaths globally.^[^ ^16^ ^]^ The sensitivities of the MagMQD@Si^+^‐based ICA for the simultaneous detection of *P. aeruginosa*, *S. pneumoniae*, and *S. typhi* are 8, 31, and 40 cells mL^−1^, respectively, and are approximately 5.2 times those of the traditional double sandwich‐based fluorescent ICA and at least 25 times those of AuNP‐based ICAs. Our proposed method exhibits good accuracy and reliability in the detection of simulated clinical and environmental samples (throat swab and lake water) and 30 real clinical specimens from patients with bacterial infections, therefore showing great potential for the rapid identification of pathogens and early diagnosis of bacterial infections in the field.

## 2 Results and Discussion

### 2.1 Detection Principle of the Electropositive MagMQD@Si^+^‐Based ICA for Bacteria

**Scheme 1:**
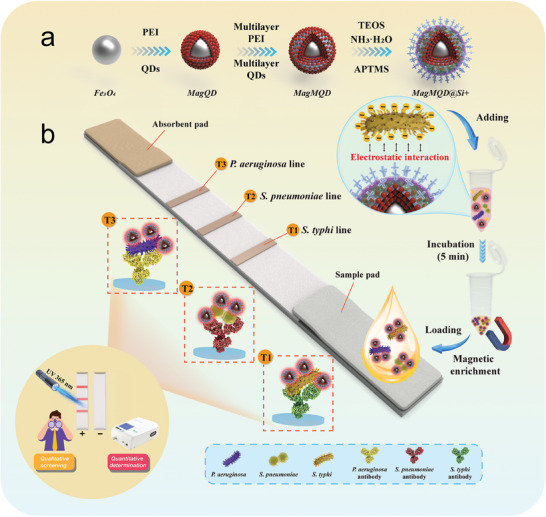
Schematic of the a) fabrication of the electropositive MagMQD@Si^+^ probe and b) principle of the antibody‐free nanoprobe‐based ICA for the simultaneous detection of three target bacteria.

**Scheme** **1** illustrates the principle and workflow of the universal ICA platform based on the antibody‐free nanoprobe MagMQD@Si^+^ for the simultaneous diagnosis of *S. typhi*, *S. pneumoniae*, and *P. aeruginosa*. The multifunctional MagMQD@Si^+^ probe is ingeniously designed and easily fabricated via the continuous coating of multilayers of QDs (≈12 nm) and one layer of an amino‐modified SiO_2_ shell on the Fe_3_O_4_ core (≈200 nm). This probe possesses superior fluorescence property, strong magnetic responsiveness, monodispersity, and high surface positive charges (Scheme 1a). Our multiplex ICA system based on the MagMQD@Si^+^ probe is a typical capture‐detection two‐step system, as depicted in Scheme 1b. First, the MagMQD@Si^+^ probes are added to the tested sample for the broad‐spectrum binding of bacteria through electrostatic interactions between the electropositive probe and electronegative bacterial cell and rapidly enriched from the solution by using an applied magnetic field. Subsequently, the collected MagMQD@Si^+^ complexes are resuspended with running buffer and then dropped onto the sample pad of the ICA strip to start immunochromatographic detection. Three kinds of antibacterial antibodies (anti‐*S. typhi*, anti‐*S. pneumoniae*, and anti‐*P. aeruginosa*) are separately sprayed on the NC membrane to construct three T lines (T1, T2, and T3) for the simultaneous detection of the three target bacteria. When the tested samples contain the target bacteria, the formed probe‐bacterial complexes flow through the entire NC membrane and are captured by the corresponding T lines via the specific binding of the antibody and antigen (bacterium). In this case, distinct fluorescence signals on the T lines of the test strip could be clearly observed. If the sample is free of the target pathogens, the MagMQD@Si^+^ complexes are not specifically recognized by the T lines and flow through the NC membrane. Therefore, no observable signals are generated in the T zones. After the chromatographic process is completed, the fluorescence intensity of each test line could be directly observed by the naked eye under UV light (qualitative diagnosis) or precisely measured with a portable fluorescence analyzer to achieve the quantitative detection of pathogens.

### 2.2 Characterization of the Electropositive MagMQD@Si^+^ Probe

**Figure 1:**
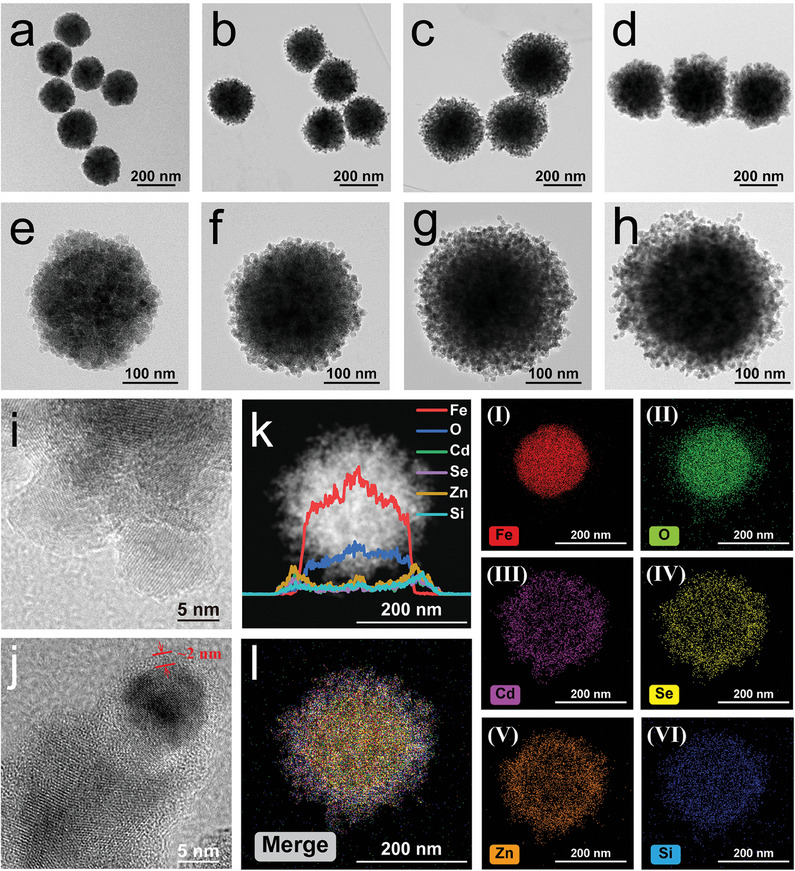
Morphological characterization of the MagMQD@Si^+^ probe. TEM images of a) Fe_3_O_4_ (200 nm), b) MagQD, c) MagMQD, and d) MagMQD@Si^+^ nanoprobes. The corresponding high‐resolution images of their individual particles are shown in (e–h). Locally amplified TEM images of the outer shells of i) MagMQD and j) MagMQD@Si^+^. k) EDS elemental line scan and l) elemental mapping results of the MagMQD@Si^+^ nanoprobe. I–VI) show the distribution of Fe, O, Cd, Se, Zn, and Si elements on MagMQD@Si^+^.

**Figure 2:**
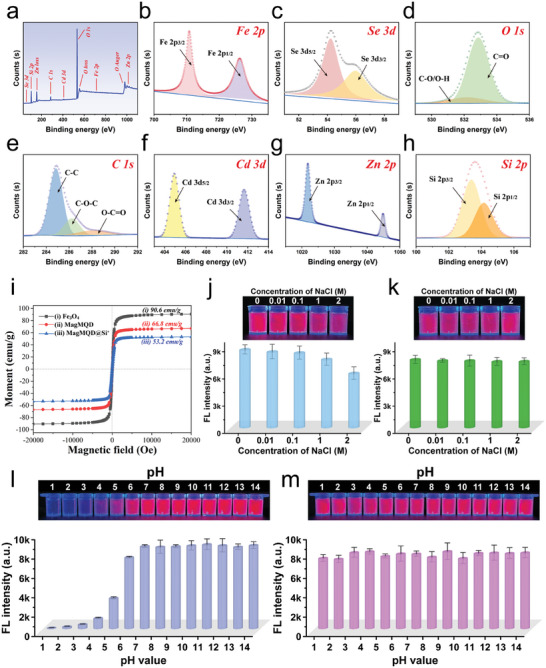
a) Wide‐scan XPS spectra and b–h) corresponding high‐resolution b) Fe 2p, c) Se 3d, d) O 1s, e) C 1s, f) Cd 3d, g) Zn 2p, and h) Si 2p spectra of MagMQD@Si^+^. i) Magnetic hysteresis loops of Fe_3_O_4_, MagMQD, and MagMQD@Si^+^. Stability of j) MagMQD and k) MagMQD@Si^+^ at various salt concentrations. Photographs and corresponding fluorescence intensity of aqueous solutions of l) MagMQD and m) MagMQD@Si^+^ at different pH values. The error bar represents the standard deviation calculated from the five sets of samples (*n* = 5).

We first confirmed the detailed nanostructure of the fabricated MagMQD@Si^+^ by using transmission electron microscopy (TEM). **Figure** **1**a shows that the fabricated Fe_3_O_4_ MNPs have good dispersibility and a uniform particle size of approximately 200 nm. After modification with PEI, the surface potential of the resulting Fe_3_O_4_/PEI MNPs has immediately changed from negative (−21.4 mV) to positive (15.1 mV), providing strong positive charges for the electrostatic adsorption of carboxylated QDs (Figure S1, Supporting Information). A large number of CdSe/ZnS‐COOH QDs are effectively adsorbed onto the surface of Fe_3_O_4_/PEI MNPs under ultrasound treatment, and the formed MagQD exhibits a rough surface with numerous small dots (Figure 1b). By using the same strategy (PEI‐mediated electrostatic adsorption), Mag@MQD with multiple layers of dense QDs can be easily synthesized by repeating PEI modification and QD coating.^[^ ^17^ ^]^ The resulting Mag@MQD MNPs are loaded with numerous QDs on their surfaces and demonstrate a typical core–porous shell structure (Figure 1c). The high‐resolution TEM (HRTEM) images of single MNP (Figure 1e–g) illustrate that the density and amount of QDs on the surface of the magnetic nanostructures increase with the continuous adsorption of multilayer PEI/QDs, suggesting that the luminous performance of MagMQD is continuously enhanced. A thin amino‐embedded SiO_2_ shell is directly coated on the surface of MagMQD through the co‐condensation of tetraethyl orthosilicate (TEOS) with APTMS in an alkaline environment.^[^ ^18^ ^]^ TEM images (Figure 1d,h) reveal that the obtained MagMQD@Si^+^ MNPs possess a complete thin SiO_2_ shell and still maintain good dispersion. The surface details of MagMQD and MagMQD@Si^+^ are shown in Figure 1i,j, respectively. These figures illustrate that the thickness of the coated electropositive SiO_2_ shell is approximately 2 nm. The zeta potential of the final product has dramatically increased from −9.8 mV to 27.4 mV (Figure S1, Supporting Information), indicating the successful coating of the amino‐modified SiO_2_ shell on the MagMQD surface. We further employed EDS element line scanning and element mapping to investigate the element distribution on the MagMQD@Si^+^ MNPs. The results of EDS element line scanning reveal the presence of multiple layers of QDs and the SiO_2_ shell outside the Fe_3_O_4_ core (Figure 1k). Meanwhile, the element mapping images illustrate the hierarchical distribution of Fe, O, Cd, Se, Zn, and Si atoms in the MagMQD@Si^+^ nanostructure (Figure 1l). We studied the surface compositions of MagMQD@Si^+^ by using X–ray photoelectron spectroscopy (XPS). The wide‐scan XPS results (**Figure** **2**a) and high‐resolution XPS spectra of Fe 2p, Se 3d, O 1s, C 1s, C 1s, Cd 3d, Zn 2p, and Si 2p (Figure 2b–h) demonstrate the abundance of the surface elements of MagMQD@Si^+^. These results are in line with expectations and verify that no other impurities are present in the multilayer nanocomposites. They confirm the successful fabrication of a multilayer magnetic QD nanocomposite with a thin SiO_2_ shell.

The amino‐modified SiO_2_ shell of MagMQD@Si^+^ has three notable functions in our detection system: i) It provides a strong positive electrical property for capturing bacteria. ii) It improves the fluidity of the large magnetic QD probes on the test strip. iii) It protects inner QDs in complex environments. We chose the thin amino‐SiO_2_ shell (≈2 nm) to coat MagMQD because it has a minimal effect on the magnetic responsiveness of the magnetic nanoprobe and could provide sufficient positive charges. Figure 2i shows that the saturation magnetization (MS) value of MagMQD@Si^+^ with a thin SiO_2_ shell remains high (53.2 emu g^−1^). Notably, the thickness of the SiO_2_ shell seriously affects the performance of electropositive probes. Two kinds of MagMQD@Si^+^ with different SiO_2_ thickness (2 and 40 nm) are prepared and compared (Figure S2a–d, Supporting Information). The results in Figure S2e,g (Supporting Information) illustrate that when the MagMQD probe is coated with a thick SiO_2_ shell (≈40 nm), its zeta potential increases to 38.5 mV and its MS value decreases to 28.2 emu g^−1^. We found that MagMQD@Si^+^ (2 nm SiO_2_ shell) could be fully collected from the sample solution (1 mL) in 1 min (Figure S2f, Supporting Information). By contrast, a considerable amount of MagMQD@Si^+^ with a thick SiO_2_ shell (≈40 nm) could not be magnetically collected in 1 min (Figure S2f, Supporting Information), indicating that MagMQD@Si^+^ with a thin SiO_2_ shell is more suitable as an ideal and efficient magnetic separation tool. In addition, the photographs and fluorescence spectra in Figure S3 (Supporting Information) show that the fluorescence intensity of the magnetic probes increases with the continuous accumulation of QDs, and the coating of the thin electropositive Si shell (<2 nm) has a negligible influence on the fluorescence intensity of MagMQD. Moreover, the MagQD nanocomposites with multiple QD shells (MagMQD and MagDQD) exhibited higher luminescence intensities than MagQD with enhancement abilities of 1.80 and 3.44 times, respectively, by comparing the intensities of the maximum emission peaks at 630 nm (Figure S3b, Supporting Information). These results verified that coating more than two layers of QD shells onto the Fe_3_O_4_ core can greatly enhance fluorescence ability of electropositive probes. The reproducibility of the coating method is next validated. As displayed in Figure S6 (Supporting Information), 10 batches of MagMQD@Si^+^ probes exhibited stable zeta potential values with relative standard deviations (RSDs) of less than 4.10%. These results indicated the proposed fabrication method for MagMQD@Si^+^ probe is a highly repeatable approach. Next, we roughly calculated the number of CdSe/ZnS QDs (12 nm) loaded on the surface of the MagMQD, confirming that multi‐layered QD shell encapsulation greatly increases the loading capacity of QDs onto the electropositive nanostructure (Section S2 and Figure S4, Supporting Information).

We evaluated the protective effect of the SiO_2_ shell on our proposed magnetic QD probe by testing the stability of MagMQD@Si^+^ in complex samples. Figure 2k,m illustrates that the dispersibility and fluorescence intensity of our MagMQD@Si^+^ probe are stable in high‐concentration salt (10–2000 mm NaCl) and aqueous solutions over a wide range of pH 1–14 within 12 h. However, the fluorescence intensity of common MagMQD starts to decline remarkably when the NaCl concentration exceeds 1000 mm (Figure 2j). In addition, the fluorescence stability of the MagMQD solution decreases after treatment with acidic solutions (pH 1–4) (Figure 2l). Moreover, the MagMQD@Si^+^ probe has high magnetic responsiveness and stable fluorescence intensity after 60 d of storage (Figure S5, Supporting Information). These results verify that the SiO_2_ shell coating greatly improves the stability of MagMQD@Si^+^ in harsh environments and effectively protects the fluorescence of inner QDs from interference or quenching.

### 2.3 Evaluation of the Bacterial Capture and Detection Ability of Electropositive MagMQD@Si^+^

**Figure 3:**
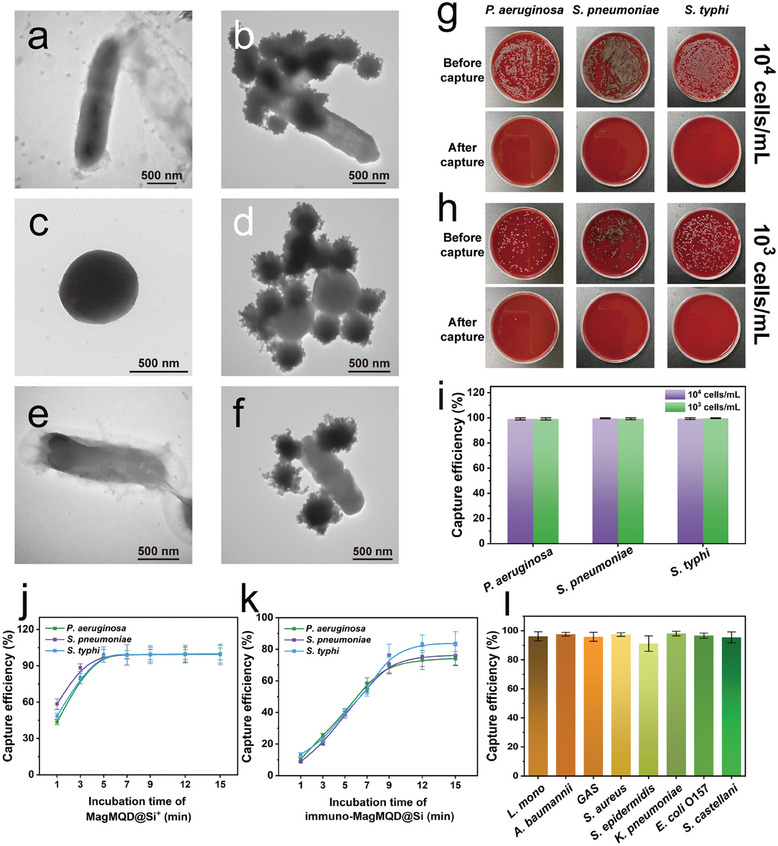
a‐i) Verification of the capture ability of MagMQD@Si^+^ for three target pathogens. TEM images of a) *P. aeruginosa*; c) *S. pneumoniae*; e) *S. typhi*; and the formed b) MagMQD@Si^+^‐*P. aeruginosa*, b) MagMQD@Si^+^‐*S. pneumonia*, and f) MagMQD@Si^+^‐*S. typhi* complexes. Plate culture results showing the bacterial quantities in the sample solution before and after MagMQD@Si^+^ capture with bacterial concentrations of g) 10^4^ and h) 10^3^ cells mL^−1^, respectively. i) Calculated capture efficiency of the MagMQD@Si^+^ probe for the three target bacteria. Capture efficiency of j) MagMQD@Si^+^ probe and k) immuno‐MagMQD@Si versus incubation time. l) Calculated capture efficiency of the MagMQD@Si^+^ probe for other eight common pathogenic bacteria. The error bar represents the standard deviation calculated from the five sets of samples (*n* = 5).

**Figure 4:**
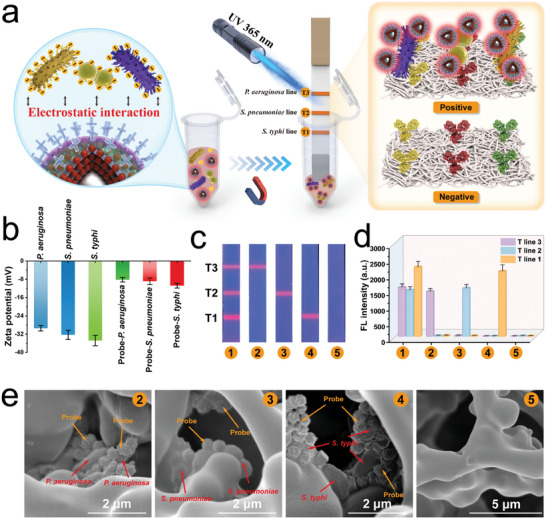
a) Schematic illustration of multiplexed fluorescent ICA based on electropositive MagMQD@Si^+^ probe for bacteria detection. b) Zeta potentials of MagMQD@Si^+^, bacteria, and the formed MagMQD@Si^+^‐bacterium complexes. Selectivity of the MagMQD@Si^+^‐based multiplex ICA for the three target bacteria: c) fluorescence images of the test strips for the detection of d) a mixture of the three target bacteria, (2) *P. aeruginosa*, (3) *S. pneumoniae*, (4) *S. typhi*, and (5) the blank control. e) SEM images of the T1 line on strip 2 (*P. aeruginosa* group), T2 line on strip 3 (*S. pneumoniae* group), T3 line on strip 4 (*S. typhi* group), and T1 line on strip 5 (blank control). The error bar represents the standard deviation calculated from the five sets of samples (*n* = 5).

Herein, we designed the electropositive MagMQD@Si^+^ for the broad‐spectrum capture of different bacteria in sample solutions on the basis of the electrostatic interactions between the amino‐SiO_2_ shell and bacterial cells. We systematically assessed the binding and capture abilities of our electropositive magnetic probe by directly reacting MagMQD@Si^+^ with bacterial samples. **Figure** **3**a,c,e shows the typical morphologies of *P. aeruginosa*, *S. pneumoniae*, and *S. typhi*, respectively. After 10 s of incubation with MagMQD@Si^+^, the surfaces of the three target bacteria have bound to several large spherical probes, and the formed MagMQD@Si^+^‐bacterium complexes can be rapidly enriched under an external magnetic field (Figure 3b,d,f). Meanwhile, the zeta potential values of the three bacteria have changed from highly electronegative (−34.8 –−29.4 mV) to weakly electronegative (−10.8–−8.2 mV) after the formation of the probe‐bacterium complexes, clearly revealing that the strong and broad‐spectrum binding ability of MagMQD@Si^+^ to bacteria does originate from electrostatic adsorption (**Figure** **4**b).

Next, we validated the capture efficiency of our MagMQD@Si^+^ probe for the three pathogens by using the plate counting method. The incubation time for bacterial capture was fixed to 5 min. The colony formation on agar plates before and after MagMQD@Si^+^ captured 10^4^ and 10^3^ cells mL^−1^ *P. aeruginosa*, *S. pneumoniae*, and *S. typhi* samples are shown in Figure 3g,h, respectively. These results indicate that the capture efficiency of our MagMQD@Si^+^ probe for the three target bacteria exceeds 99.0% (Figure 3i). Moreover, our electropositive probe exhibits time‐dependent behavior in the magnetic enrichment of pathogens and can reach its highest capture efficiency within 5 min (Figure 3j). Notably, MagMQD@Si^+^ possesses superior bacterial capture ability over antibody‐modified MagMQD@Si probes. Figure 3k reveals that the highest capture efficiencies of the conventional immuno‐MagMQD@Si probe for the three kinds of bacteria are 74.1%–83.7% and that the optimal incubation time for bacterial capture is approximately 12 min. These results verify that the binding ability and reaction rate of the electropositive SiO_2_ shell‐mediated electrostatic interaction are considerably higher than those of the antibody‐mediated antigen–antibody immune response for bacterial cells. These characteristics can effectively improve magnetic enrichment ability and reduce the capture time of magnetic QD probes, thus enhancing the detection sensitivity and efficiency of our proposed ICA platform. Notably, MagMQD@Si^+^ has broad‐spectrum capture ability for other common pathogens, including *Listeria monocytogenes* (*L. monocytogenes*), group A *Streptococcus* (*GAS*), *Acinetobacter baumannii* (*A. baumannii*), *Staphylococcus aureus* (*S. aureus*), *Staphylococcus epidermidis* (*S. epidermidis*), *Klebsiella pneumoniae* (*K. pneumoniae*), *Escherichia coli* (*E. coli*) O157 and *Shigella castellani* (*S. castellani*). As shown in Figure 3l, the capture efficiencies of MagMQD@Si^+^ for other seven common pathogens are also higher than 91% after 5 min of enrichment, indicating the electrostatic‐mediated bacterial capture method is universal and practical for pathogens. Therefore, the electropositive MagMQD@Si^+^ can act as a universal enrichment tool for the separation and detection of different bacteria.

**Figure 5:**
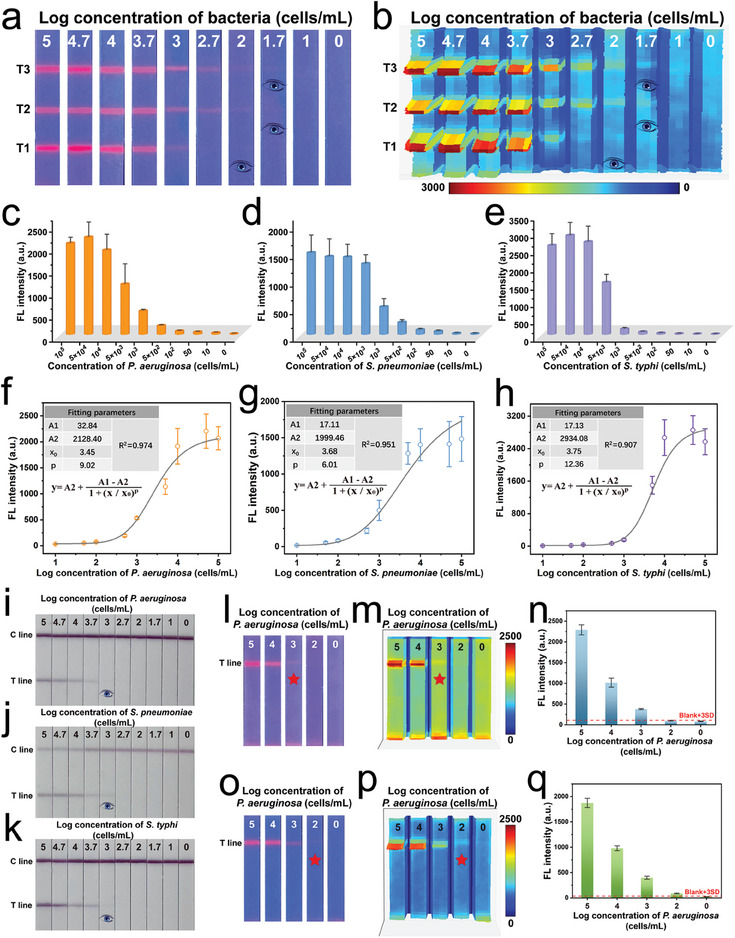
Analytical performance of the fluorescent MagMQD@Si^+^‐based ICA in detecting multiple pathogenic bacteria. a) Photograph and b) 3D heatmaps of fluorescence intensities on ICA strips for the simultaneous detection of *P. aeruginosa*, *S. pneumoniae*, and *S. typhi*. Detailed fluorescence intensities on the three T lines (T3, T2, and T1) corresponding to varying concentrations of c) *P. aeruginosa*, d) *S. pneumoniae*, and e) *S. typhi*. Calibration curves for f) *P. aeruginosa*, g) *S. pneumoniae*, and h) *S. typhi* based on the fluorescence intensities on T lines. Detection results of the AuNP‐based ICA for i) *P. aeruginosa*, j) *S. pneumoniae*, and k) *S. typhi*. l–q) Comparison between the performances of the electropositive MagMQD^+^ and MagMQD@Si^+^ probes on ICA strips. l) Photograph, m) 3D heatmaps, and n) fluorescence intensity values of the MagMQD^+^‐based ICA for *P. aeruginosa* detection. o) Photograph, p) 3D heatmaps, and q) fluorescence intensity values of the MagMQD@Si^+^‐based ICA for *P. aeruginosa* detection. The error bar represents the standard deviation calculated from the five sets of samples (*n* = 5).

Encouraged by the distinct advantages, including superior fluorescence property and high stability and capture ability, of our proposed electropositive magnetic probe, we employed MagMQD@Si^+^ in multiplex ICA for the on‐site and simultaneous diagnosis of common pathogens. Schematic illustration of multiplexed fluorescent ICA based on electropositive MagMQD@Si^+^ probe for bacteria detection was shown in Figure 4a. First, we evaluated the selectivity of the MagMQD@Si^+^‐based ICA by setting five groups of samples containing different concentrations of *P. aeruginosa*, *S. pneumoniae*, and *S. typhi* (1: 10^4^/10^4^/10^4^ cells mL^−1^; 2: 10^4^/0/0 cells mL^−1^; 3: 0/10^4^/0 cells mL^−1^; 4: 0/0/10^4^ cells mL^−1^; 5: 0/0/0 cells mL^−1^). Figure 4c,d shows the photographs of the test ICA strips and corresponding fluorescence intensity on the three T lines, respectively. Apparently, no detectable fluorescence signal could be observed on the test strip for the negative group, whereas the positive samples containing the target pathogens have produced bright red fluorescence on the corresponding T zones, revealing the absence of cross‐reactivity between the three independent detection channels of our MagMQD@Si^+^‐based ICA. We employed a scanning electron microscope (SEM) to investigate the internal morphology of the T lines of the test strips. Figure 4e‐2–5 show the SEM images of the corresponding T zones for the detection of *P. aeruginosa*, *S. pneumoniae*, *S. typhi*, and the negative sample, respectively. Numerous MagMQD@Si^+^‐bacterium complexes are observed in the fiber pores of the T lines for the positive samples, whereas no MagMQD@Si^+^ labels or bacterial cells could be found in the T zone of the negative control, indicating that the strong fluorescent response is derived from the formation of the MagMQD@Si^+^‐bacterium‐antibody sandwich structure on the T line. On the basis of the above results, we confirmed the excellent multichannel detection capability of the MagMQD@Si^+^‐based fluorescent ICA.

Subsequently, we further optimized the key parameters of the MagMQD@Si^+^‐based fluorescent ICA platform to increase sensitivity and stability for bacterial detection. The optimization focused on the concentration of the antibody coating on the T line (Figure S7, Supporting Information), amount of MagMQD@Si^+^ probes (Figure S8, Supporting Information), time of incubation (Figure S9a, Supporting Information), time of chromatographic reaction (Figure S9b, Supporting Information), and composition of the sample loading solution (Figure S10, Supporting Information). The optimization processes are discussed in Section S3 (Supporting Information). All the optimized parameters were used in subsequent experiments. Notably, we confirm that 5 min of magnetic capture process can reach the highest capture efficiency of MagMQD@Si^+^ probe for three target bacteria (Figure 3j), whereas 10 min of chromatographic time is enough for MagMQD@Si^+^‐based ICA for bacteria detection (Figure S9, Supporting Information). These results indicate the proposed MagMQD@Si^+^‐ICA enables fast detection of bacteria within 15 min, including a 5 min magnetic capture process and a 10 min reaction time on the test strip.

### 2.4 Evaluation of the Detection Performance of the MagMQD@Si^+^‐Based ICA Platform

We first evaluated the analytical performance (sensitivity, quantitative ability, and detection range) of our proposed ICA method in mixed bacterial samples containing different concentrations (0–10^5^ cells mL^−1^) of *P. aeruginosa*, *S. pneumoniae*, and *S. typhi*. We determined the original concentrations of the three target bacteria by using the plate counting method (Figure S11, Supporting Information), which ensured the accuracy of the concentration of the prepared bacterial samples. **Figure** **5**a,b show the photographs and corresponding fluorescence intensities on whole test strips for the three target pathogens, respectively. In these figures, T1, T2, and T3 show the detection results for *S. typhi*, *S. pneumoniae*, and *P. aeruginosa*, respectively. As expected, the intensity of the fluorescence signal on the T1/T2/T3 lines is positively proportional to the concentrations of *S. typhi*/*S. pneumoniae*/*P. aeruginosa* in the samples. The weakest signals on the T lines visible to the naked eye are found at the *P. aeruginosa*, *S. pneumoniae*, and *S. typhi* concentrations of 50, 50, and 100 cells mL^−1^, respectively. The detailed fluorescence intensity values of the three T lines on each strip were directly read by using a portable fluorescence immunoassay analyzer (Figure 5c–e). The generated fluorescence signals on the T lines (T3 and T1) corresponding to *P. aeruginosa* and *S. typhi* are directly proportional to the bacterial concentrations in the samples within the range of 50–5 × 10^4^ cells mL^−1^, whereas the fluorescence intensity values of the T2 line for *S. pneumoniae* is directly proportional to the bacterial concentrations in the samples within the range of 50–10^5^ cells mL^−1^. We performed calibration curve calculations on the basis of the measured fluorescence signals for the quantitative analysis of the three target pathogens by using the MagMQD@Si^+^‐based ICA. Figure 5f–h shows the obtained calibration curves and corresponding four‐parameter logistic equations for *P. aeruginosa*, *S. pneumoniae*, and *S. typhi*, respectively. The limit of detection (LOD) values of the proposed ICA for *P. aeruginosa*, *S. pneumoniae*, and *S. typhi* are 8, 31, and 40 cells mL^−1^, respectively, with the correlation coefficients (*R* ^2^) values exceeded 0.9, as calculated using the typical IUPAC method (LOD = mean fluorescence intensity of the blank control plus three times the value of the standard deviation of the blank control).^[^ ^19^ ^]^ The MagMQD@Si^+^‐based ICA showed slightly different sensitivities for the three target pathogens, which can be attributed to the differing activities of the antibodies used.^[^ ^20^ ^]^ Such low LOD values of our proposed ICA technology reach the level of qPCR (gold standard) for bacteria detection. Moreover, compared with the conventional AuNP‐based ICA method, our electropositive nanoprobe‐mediated ICA exhibits superior performance, including considerably higher sensitivity, wider detection range, better quantitative ability, and more testing channels. The results in Figure 5i–k show that the visual detection limits of our AuNP‐based ICA strips for *P. aeruginosa*, *S. pneumoniae*, and *S. typhi* are approximately 10^3^ cells mL^−1^ with the dynamic detection range of 10^5^–10^3^ cells mL^−1^. Compared with that of AuNP nanoprobes, the use of our MagMQD@Si^+^ probe has improved the sensitivity and detection range of our ICA platform for the three target bacteria by 25–125 times and approximately two orders of magnitude. Notably, the high sensitivity of the established electropositive probe‐ICA mainly comes from the use of MagMQD@Si^+^ probe with multilayer QD shells. We have verified that the detection sensitivity of proposed ICA using MagMQD@Si^+^ is improved by about 44 times and 8.5 times compared to those of using MagQD@Si^+^ and MagDQD@Si^+^ probes, respectively (Figure S12, Supporting Information). We also compared the detection capability of our electropositive MagMQD@Si^+^ and conventional immuno‐MagMQD@Si probe by detecting the same group of bacterial samples. We fabricated the immuno‐MagMQD@Si probe for *S. typhi* via the conjugation of specific antibodies to the TEPSA‐modified SiO_2_ shell through EDC/NHS chemistry (Section S1.3, Supporting Information). Figure S13a,c (Supporting Information) shows the photographs of the immuno‐MagMQD@Si‐ and MagMQD@Si^+^‐based ICAs for *S. typhi* detection. The MagMQD@Si^+^ probe generates brighter fluorescence intensity on the T line than the immuno‐MagMQD@Si probe, achieving a fivefold improvement in the visual sensitivity of the ICA method. We constructed the calibration curves of the two ICA methods by using the detailed fluorescence signals on the corresponding T lines, as displayed in Figure S13b,d (Supporting Information). Accordingly, we calculated the LOD for *S. typhi* of the immuno‐MagMQD@Si‐based ICA to be 302 cells mL^−1^, which is 5.2 times higher than that of the MagMQD@Si^+^‐based strip (58 cells mL^−1^). These results indicate that our electropositive nanoprobe‐based ICA possesses higher detection sensitivity than the traditional double antibody sandwich ICA strip for bacteria and exhibits high specificity and stability, thus suggesting its strong potential for clinical diagnostics and POCT. The superior performance of our method can be attributed to the higher capture ability of our electropositive nanoprobe for bacteria than that of antibody‐modified nanoprobes. More importantly, compared to previously reported works, our research not only elucidates the universality and stability of electropositive probes on ICA for the first time, but also significantly enhances sensitivity and detection throughput for bacteria via ICA technology (Table S1, Supporting Information). The combination of electropositive probes and the multiplex ICA strip will effectively improve the limitations of existing immunosensor for bacteria detection.

Subsequently, we conducted a contrast experiment to demonstrate the effect of the SiO_2_ shell on the detection performance of our electropositive nanoprobe‐based ICA method. We prepared a MagMQD^+^ probe by directly coating cationic PEI on the surface of MagMQD. The characterization data are shown in Figure S14 (Supporting Information). When we applied MagMQD^+^ as the electropositive nanoprobe, the test strips exhibit good feasibility for detecting bacteria, with vLOD and LOD values of 10^3^ cells mL^−1^ (Figure 5l–n). However, distinct background signals are clearly observed on the NC membrane of the MagMQD^+^‐based ICA strip. These signals can mask weak positive signals on the T line in the detection of low concentrations of bacteria and thus decrease detection sensitivity. Encouragingly, our SiO_2_‐coated electropositive nanoprobe (MagMQD@Si^+^) has almost no background signal on the whole test strip and effectively improves the sensitivity for pathogen diagnosis by at least 10 times (Figure 5o–q). In addition, the comparison of the flow properties of MagMQD@Si^+^ and MagMQD^+^ probes on the test strips is presented in the Figure S15 (Supporting Information), which confirms that MagMQD@Si^+^ has better fluidity and can improve the dispersibility of formed bacteria‐probe complexes. From the above findings, we can conclude that coating of a SiO_2_ shell not only benefits the broad‐spectrum capture of bacteria but also improves the liquidity and detection performance of our electropositive probe on the test strip.

**Figure 6:**
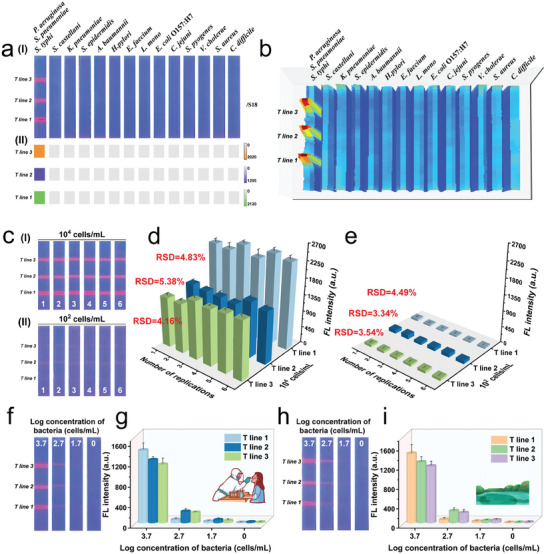
Evaluation of the specificity of the MagMQD@Si^+^‐based ICA: a) Test strip photographs (I) and corresponding heatmaps (II) of fluorescence intensities on the three T lines and b) fluorescence mapping images of whole ICA strips for target pathogens and nontarget bacteria. Assessment of the repeatability of the proposed assay: c) photographs and d–e) average fluorescence intensities of the three T lines corresponding to d) 10^4^ and e) 10^2^ cells mL^−1^ bacteria. Evaluation of the performance of the MagMQD@Si^+^‐based ICA for bacterium‐spiked complex samples: photograph of f) test strips and g) corresponding fluorescence intensity on the T lines for throat swab samples; photograph of h) test strips and i) corresponding fluorescence intensity on the T lines for lake water samples. The error bar represents the standard deviation calculated from the five sets of samples (*n* = 5).

We then evaluated the specificity of our proposed MagMQD@Si^+^‐based ICA. We detected 13 other common pathogenic bacteria, namely, *Shigella castellani*, *Klebsiella pneumoniae*, *Staphylococcus epidermidis*, *A. baumannii*, *Helicobacter pylori*, *Enterococcus faecalis*, *L. monocytogenes*, *E. coli* O157:H7, *Campylobacter jejuni*, *Streptococcus pyogenes*, *Vibrio cholerae*, *S. aureus*, and *Clostridioides difficile*, by using our established method. The results are shown in **Figure** **6**a,b. The fluorescence images and fluorescence scan results of the ICA strips reveal that only the tested samples containing the target bacteria (*P. aeruginosa*, *S. pneumoniae*, and *S. typhi*) could generate specific signal responses on the corresponding T zones, whereas all 13 nontarget pathogens do not generate any fluorescence intensity on the MagMQD@Si^+^‐ICA strips. These results indicate the high specificity of the proposed ICA method for target bacteria. Moreover, the selectivity of the electropositive probe‐based ICA technique was tested by detecting different mixed bacterial samples. The results in Figure S16 (Supporting Information) clearly demonstrated the presence of non‐target bacteria in the sample will not interfere with the accuracy of each independent T line, indicating the good selectivity of MagMQD@Si^+^‐ICA for target pathogens. These results confirm that the combination of the antibody‐immobilized test strip and electropositive nanoprobe can guarantee the high specificity of our proposed ICA platform for its target bacteria. Additionally, we investigated the reproducibility of our proposed method by testing six batches of independently prepared bacterial samples containing 10^4^ and 10^2^ cells mL^−1^ *P. aeruginosa*/*S. pneumoniae*/*S. typhi*. The results for all six groups show that the tested ICA strips based on MagMQD@Si^+^ generate homogeneous fluorescence signals for bacterial samples with the same concentration, with coefficient of variation (CV) ≤ 5.38% (Figure 6c–e). These results demonstrate that our established system has good reliability and stability. The reproducibility of the preparation method for electropositive MagMQD@Si^+^‐ICA system has been evaluated and the results are shown in Figure S17 (Supporting Information). In addition, the MagMQD@Si^+^‐ICA strips exhibit stable fluorescence intensity of the test lines for bacteria detection after 60 d of storage, indicating the excellent long‐term stability of proposed assay (Figure S18, Supporting Information).

Given the efficient enrichment ability of MagMQD@Si^+^, the proposed ICA method can achieve the rapid capture of target bacteria from complex samples and their accurate detection on the test strips. We spiked typical clinical throat swab and environmental samples (lake water) with different concentrations of *P. aeruginosa*/*S. pneumoniae*/*S. typhi* to simulate bacterium‐contaminated real samples. The concentration gradients of the three target pathogens in the two kinds of real samples were set to 5000/5000, 500/500, 50/50, and 0/0 cells mL^−1^. Figure 6f–i illustrates that the MagMQD@Si^+^‐based ICA method works well on throat swab and lake water samples and generates stable fluorescence signal intensities on the corresponding T zones similar to those generated in PBS samples (Figure 5a). By inputting the measured fluorescence signals of the T zones into the pre‐established standard calibration curves (Figure 5f–h), we found that the average recovery rates of our MagMQD@Si^+^‐based ICA for the three target bacteria range from 90.82% to 107.69% with relative standard deviations (RSDs) of all less than 10% (Table S2, Supporting Information). These results suggest that our ICA method based on MagMQD@Si^+^ can stably operate in complex samples and exhibit accurate quantitative ability for bacteria.

### 2.5 Application of the MagMQD@Si^+^‐Based ICA for Real Clinical Specimens

**Figure 7:**
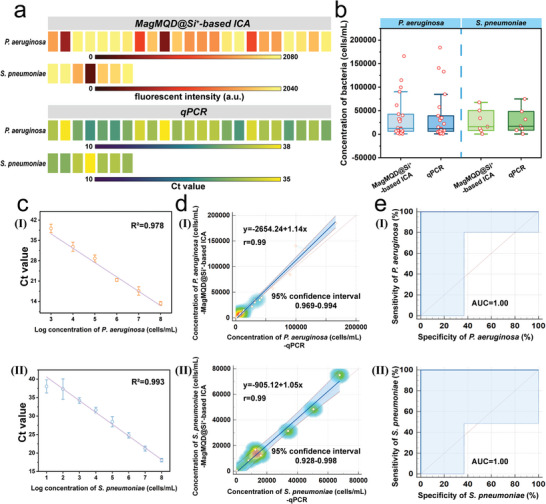
Detection results of the MagMQD@Si^+^‐based ICA for real clinical respiratory specimens. a) T line fluorescence intensity on MagMQD@Si^+^‐ICA strips (I) and CT value of qPCR (II) for the detection of 23 *P. aeruginosa*–positive sputum samples, 7 *S. pneumoniae*‐positive sputum samples, and 10 target pathogen‐negative sputum samples. b) Box diagram of the MagMQD@Si^+^‐ ICA and qPCR for the detection of *P. aeruginosa* and *S. pneumoniae*. c) Standard curve of qPCR for *P. aeruginosa* (I) and *S. pneumoniae* (II) detection. d) Deming regression analysis of *P. aeruginosa* (I) and *S. pneumoniae* (II) detection by the MagMQD@Si^+^‐based ICA and qPCR. e) ROC curve of the negative and positive prediction models for the diagnosis of *P. aeruginosa* (I) and *S. pneumoniae* (II) infections based on the MagMQD@Si^+^‐based ICA. The error bar represents the standard deviation calculated from the five sets of samples (*n* = 5).

Finally, we applied our electropositive nanoprobe‐mediated ICA technique in the diagnosis of real clinical respiratory specimens from patients with bacterial lung infections. We collected 23 *P. aeruginosa*‐positive sputum samples, 7 *S. pneumoniae‐*positive sputum samples, and 10 target pathogen‐negative sputum samples from the Clinical Laboratory of Guangdong Provincial People's Hospital under the guidance of the hospital's ethics committee (approval number: KY2024‐678‐02). We verified these clinical respiratory sputum samples via realtime fluorescence quantitative PCR (qPCR) and sputum smear before ICA detection (Sections S4.1–S4.3 and Table S3, Supporting Information). **Figure** **7**a shows the detection results (T line fluorescence intensity and Ct value) of the MagMQD@Si^+^‐based ICA method and qPCR for samples positive for *P. aeruginosa* and *S. pneumoniae*. As shown in Figure 7c, we plotted the standard curve of the qPCR method on the basis of the relationship between i) *P. aeruginosa* and ii) *S. pneumoniae* concentration and Ct values. We inputted the T line fluorescence intensities and Ct values of the 23 *P. aeruginosa*‐positive sputum samples and 7 *S. pneumonia*‐positive sputum samples into the standard curves of the MagMQD@Si^+^‐based ICA and qPCR methods established as shown in Figures 5f,g and 7c to obtain the bacterial concentrations detected by the two methods (Table S4, Supporting Information). In the detection of the same target bacteria, the box diagrams of the MagMQD@Si^+^‐based ICA and qPCR methods show similar shapes, preliminarily indicating that the detection results of the two methods are consistent (Figure 7b). The Deming regression line in Figure 7d illustrates that *P. aeruginosa* and *S. pneumoniae* have slopes that approach 1 (*P. aeruginosa*: 1.14 and *S. pneumoniae*: 1.05) and 95% confidence intervals of 0.969–0.994 and 0.928–0.998, respectively. The ROC curves in Figure 7e further demonstrate that our method has 100% sensitivity and specificity for real clinical specimens. All the above results verify that the proposed MagMQD@Si^+^‐based ICA method has the same accuracy as qPCR but only needs 1/9 of the testing time of PCR (our MagMQD@Si^+^‐based ICA requires 20 min, whereas qPCR generally requires 3 h). Moreover, the operation of the MagMQD@Si^+^‐based ICA is simpler and faster than those of current clinical pathogen detection technologies, such as ELISA and PCR. Furthermore, considering that our electropositive nanoprobe‐based ICA technique needs only MagMQD@Si^+^ as the universal probe and a multichannel test strip (wherein one antibody‐loaded T line detects one target) for the detection of different target pathogens, the stability, cost, and antibody dependence of the proposed assay are substantially lower than those of common double‐antibody sandwich ICA methods. Therefore, our method possesses great potential for development into a universal detection platform for bacteria.

## 3 Conclusions

We report for the first time an ultrasensitive fluorescent ICA platform for bacterial diagnosis with universal and multiplex detection ability based on the broad‐spectrum capture ability of electropositive MagMQD@Si^+^ for negatively charged bacterial cells. Our newly invented antibody‐free MagMQD@Si^+^ probe consists of a large Fe_3_O_4_‐formed magnetic core (≈200 nm), multilayer QD‐formed fluorescent inner shell, and thin amino‐functionalized SiO_2_ shell layer (≈2 nm), which can provide strong magnetic responsiveness for target enrichment, superior fluorescence signals for ICA sensing, and universal binding capacity and high affinity for various pathogens. Our results clearly demonstrate that our electropositive MagMQD@Si^+^ allows the sensitive and specific diagnosis of different pathogenic bacteria in our multichannel ICA system through arbitrary combinations with the antibody‐loaded test strip, thus dramatically improving the detection performance (sensitivity, accuracy, and stability) and reducing the cost and difficulty of ICAs for the multiplex detection of bacteria. Our established ICA technique enables the highly sensitive quantitative detection of *P. aeruginosa*, *S. pneumoniae*, and *S. typhi* within 15 min with LOD values of 8, 31, and 40 cells mL^−1^, respectively. Compared with the conventional AuNP‐based colorimetric ICA, our electropositive probe–based platform for bacterial detection exhibits 25–125‐fold lower LOD and a dynamic range that is more than two orders of magnitude greater. Moreover, we validated our proposed assay on real‐world samples by detecting 40 clinical sputum samples (23 positive sputum samples from patients with *P. aeruginosa* infections, seven positive sputum samples from patients with *S. pneumoniae* infections, and 10 negative sputum samples with patients with nontarget pathogen infections). Our validation results show 100% concordance with the results of golden standards (qPCR and plate cultivation). Our findings verify that our electropositive probe‐based ICA has good application prospects in the on‐site screening and clinical diagnosis of bacterial infections.

## 4 Experimental Section

### Ethics Statement

All clinical sputum samples used in this work were collected from the clinical laboratory of Guangdong Provincial People's Hospital with the approval of the hospital's ethics committee (Approval number: KY2024‐678‐02). All human subjects voluntarily provided the samples required for this experiment after signing the informed consent form.

### Preparation of the Electropositive Probe MagMQD@Si^+^

First, multilayer magnetic QD NPs, which serve as sources of magnetic enrichment capability and fluorescence signals, must be prepared. Fe_3_O_4_ NPs with a diameter of ≈200 nm were synthesized through an improved coprecipitation method.^[^ ^21^ ^]^ The prepared Fe_3_O_4_ NPs were mixed with an aqueous solution of PEI (1.7 mg mL^−1^) and vigorously sonicated for 30 min. After being thoroughly washed with water, PEI‐modified Fe_3_O_4_ NPs (Fe_3_O_4_/PEI) were obtained. The Mag@PEI particles were then dispersed in 60 mL of QD solution (30 mg mL^−1^), allowing the QDs to adsorb electrostatically on the surfaces of the PEI‐coated magnetic beads, producing Fe_3_O_4_ NPs coated with a single layer of QDs (MagQDs). The QD‐modified magnetic beads were separated magnetically and washed to remove unbound QDs, then dispersed in fresh PEI solution (1.7 mg mL^−1^). The steps of QD adsorption, washing, and dispersal in PEI were repeated for multilayer QD self‐assembly until the desired multilayer QD‐loaded Fe_3_O_4_ NPs (MagMQD) were obtained.^[^ ^17a^ ^]^

Second, a strategy involving coating multilayer magnetic QDs (MagMQDs) with a protective amino‐modified SiO_2_ shell was employed to enhance the stability of the label‐free detection tags. MagMQD NPs, at a concentration of 10 mg mL^−1^, were dispersed in 60 mL of an ethanol‐deionized water mixture in a volume ratio of 1:1. Ultrasonication was applied to obtain a uniformly dispersed suspension. Subsequently, the suspension was slowly added with 5 mL of ammonia solution, 6 mL of TEOS and 500 µL of APTMS and then ultrasonicated for 90 min. After the reaction was completed, the product (MagMQD@Si^+^) was washed twice with deionized water and finally resuspended in 6 mL of anhydrous ethanol for further use.

### Preparation of the MagMQD@Si^+^‐Based ICA

An NC membrane was affixed onto a spray‐coating device, and detection antibodies specific to *S. typhi*, *S. pneumoniae*, and *P. aeruginosa* were sequentially sprayed on the membrane. The antibodies were meticulously applied at predetermined positions to form T1, T2, and T3. Following application, the membrane was incubated in a drying oven at 34 °C for 3 h to ensure stable antibody binding. Subsequently, the NC membrane, sample pad, and absorbent pad were adhered to a plastic backing in accordance with a predesigned layout. This assembly ensured appropriate overlapping among the components, thus maintaining the integrity and continuity of sample flow. The treated NC membrane was then precisely cut into strips 3 mm wide, resulting in individual immunochromatographic test strips. Finally, the prepared test strips were sealed in aluminum foil pouches for storage.

### Methodology for the Simultaneous Detection of *S. typhi*, *S. pneumoniae*, and *P. aeruginosa* by Using the MagMQD@Si^+^‐Based ICA

The detection methodology leverages an electropositive MagMQD@Si^+^ probe, which is characterized by positively charged SiO_2_‐coated multilayer QDs, for the simultaneous detection of *S. typhi*, *S. pneumoniae*, and *P. aeruginosa*. The concentrations of the three bacterial species used in this study were accurately quantified to be 10^8^ cells mL^−1^ based on the bacterial plate count results depicted in Figure S11 (Supporting Information). Electropositive MagMQD@Si^+^ probes were incubated for 5 min with a mixture containing varying concentrations of the three bacterial species (0–10^5^ cells mL^−1^). Following incubation, magnetic enrichment was performed to isolate probe–bacterium complexes. The enriched complexes were then resuspended in 80 µL of sample loading buffer, which consisted of 1% PBST (10 mm PBS with Tween 20), 1% milk and 1% BSA. The resuspended sample was subsequently loaded on the sample pad of the FL‐ICA strip, and chromatographic separation was allowed to proceed for 10 min. After the chromatographic reaction, the fluorescence signals on the three detection lines were observed visually for qualitative assessment. For quantitative analysis, a portable fluorescence spectrometer was employed to measure the fluorescence intensity on each detection line. The obtained fluorescence signal values were then plotted against known bacterial concentrations to construct a standard curve, enabling the quantification of bacterial presence in unknown samples.

The LOD values of the MagMQD@Si^+^‐based ICA were determined in accordance with the following principle (1):^[^ ^22^ ^]^

(1) $$\begin{array}{ccc} \mathrm{LOD} & = & \mathrm{mean}\;\mathrm{signals}\;\left(\mathrm{fluorescence}\;\mathrm{intensity}\right)\mathrm{on}\;\mathrm{the}\;T\;\mathrm{lines}\;\mathrm{of}\;\mathrm{the}\;\mathrm{blank}\;\\ & & \mathrm{control}+3\times \mathrm{standard}\;\mathrm{deviation}\;\mathrm{of}\;\mathrm{blank}\;\mathrm{intensity} \end{array}$$

Dual‐mode detection, which comprises qualitative visual observation and quantitative spectrometric measurement, ensures the rapid screening and precise quantification of the bacterial targets.

### Detection of Clinical Sputum Samples by Using the MagMQD@Si^+^‐Based ICA Method

The applicability and effectiveness of the MagMQD@Si^+^‐based ICA method were validated through the analysis of clinical sputum samples. Specifically, seven *S. pneumoniae*‐positive sputum samples, 23 *P. aeruginosa*‐positive sputum samples, and 10 nontarget pathogen infection sputum samples were collected from the Department of Laboratory Medicine of Guangdong Provincial People's Hospital. All sputum samples were liquefied with 4% NaOH for 30 min. The collected sputum specimens were directly analyzed by using the constructed MagMQD@Si^+^‐based ICA. The fluorescence intensity values on the detection lines were recorded, and the bacterial concentrations of the samples were determined by referencing a pre‐established standard curve that correlates fluorescence intensity with bacterial concentration. The formula (2) for the relevant standard curve is^[^ ^23^ ^]^

(2) $$y=A2+\left(A1-A2\right)/\left[1+\left(x/x_{0}\right)p\right]$$

where *y* is the measured fluorescence intensity on the T‐lines; the measured signal *x* is the concentration of the bacteria; *A*1 and *A*2 are the responses at minimum and maximum concentrations, respectively; *x*_0_ is the concentration of the bacteria at the midpoint between *A*1 and *A*2; and *p* is the slope parameter.

### Statistical Analysis

Origin 2024 software (OriginLab, Inc., USA) was used for creating graphs and conducting statistical analysis. Deming regression analysis was performed using MedCalc software to assess the consistency between MagMQD@Si^+^‐based ICA and qPCR. Each result in the figures was obtained from the mean ± standard deviation (SD) of five independent experiments (*n* = 5). The sample size (*n*) for each experiment is indicated in the Figure legends.

## Conflict of Interest

The authors declare no conflict of interest.

## Author Contributions

J.X.L., Z.K.L., and B.J.W. contributed equally to this work. C.W.W. and B.G. designed and managed the study. J.X.L., Z.K.L., B.J.W., Q.Y., and T.W. performed the experiments. J.X.L. and B.J.W. analyzed the experimental data. C.W.W., B.G., J.X.L., and Z.K.L. wrote the manuscript. All authors reviewed the manuscript.

## Supporting information



Supporting Information

## Data Availability

The data that support the findings of this study are available from the corresponding author upon reasonable request.
