# Supplementary material for: Electropositive Magnetic Fluorescent Nanoprobe‐Mediated Immunochromatographic Assay for the Ultrasensitive and Simultaneous Detection of Bacteria
Source: Adv Sci (Weinh). 2025 Jan 13;12(12):2412421. doi: 10.1002/advs.202412421 (PMC11948010; doi:10.1002/advs.202412421)
Supplement: Supplementary file 1 — Supporting Information [file ADVS-12-2412421-s001.docx]

Supporting Information

**Electropositive Magnetic Fluorescent Nanoprobe–Mediated Immunochromatographic Assay for the Ultrasensitive and Simultaneous Detection of Bacteria**

Jiaxuan Li ^a,b^, Zhengkang Li ^a^, Bingjie Wang ^a^, Qing Yu ^a,b^, Ting Wu ^a^, Chongwen Wang ^a,b,^ *, Bing Gu ^a,b,^ *

^a^ Department of Clinical Laboratory Medicine, Guangdong Provincial People’s Hospital (Guangdong Academy of Medical Sciences), Southern Medical University, Guangzhou, Guangdong 510000, China

^b^ School of Medicine, South China University of Technology, Guangzhou, 510006, China

*^*^*Corresponding author

Email: [wangchongwen1987@126.com](mailto:wangchongwen1987@126.com) (Chongwen Wang),

Email: gubing@gdph.org.cn (Bing Gu)

**Supporting Material Contents.**

S1. Supplementary experimental section

S2 Calculation of maximum number of QDs on the MagMQD

S3. [Optimization](javascript:;) of electropositive probe-based ICA system

S4. Detection of clinical samples

Supplement Figures S1-S18

Tables S1-S4

**S1. Supplementary experimental section**

**S1.1 Materials and chemicals**

APTMS ((3-Aminopropyl)trimethoxysilane), TEPSA ([(3-Triethoxysilyl)propyl]succinic Anhydride), Polyethylenimine (PEI) (MW 25 kDa), ferric chloride (FeCl_3_·6H_2_O), Tween 20, EDC, N-hydroxysulfosuccinimide sodium salt (NHS), and BSA were supplied by Sigma-Aldrich (USA). Carboxylated CdSe/ZnS-MPA QDs (catalog #CdSe-MPA-625) were obtained from Mesolight Inc. (Suzhou, China). Mouse monoclonal anti- *S. pneumoniae* antibodies (Catalog # ACT-prab-SP-001) were purchased from US-China Xinxin Biotechnologies, Ltd. (Wujin, Changzhou). Mouse monoclonal anti-*P. aeruginosa* antibodies (MA1-83430) were purchased from Scientific, Inc. (MA, the USA). Mouse monoclonal anti-*S. typhimurium* antibodies (catalog no. ab8274) were obtained from Abcam (Cambridge, the UK). LFIA subassemblies including the NC membrane (CN95), sample pad, absorbent pad, and plastic plate were obtained from Jieyi Biotechnology Co., Ltd. (Shanghai, China).

**S1.2 Characterization and instrumentation**

The TEM and SEM characterizations of fabricated nanofilms were carried out by using a FEI Tecnai G2 F20 electron microscope at an accelerating voltage of 200 kV and a JEOL JSM-7001F instrument operated at 10 kV, respectively. The zeta potentials and hydrodynamic sizes of the fabricated nanomaterials were detected on a Nano ZS90 Zeta Analyzer (Malvern, UK). The emission spectra of QD nanomaterials were recorded by using a USB2000þ spectrometer (Ocean Optics, USA) under excitation by a 365 nm UV lamp. The magnetic property of Fe_3_O_4_-based nanoparticles was investigated using a superconducting quantum interference device magnetometer (MPMSXL-7, USA) at 300 K. The fluorescence signals of the test (T) and control (C) lines of the MagMQD@Si^+^−based ICA strips were recorded on a commercial portable FIC-S1 fluorescence reader (365 nm excitation/610 nm emission, Suzhou Hemai, China).

**S1.3 Preparation of antibody-conjugated MagMQD@Si-based ICA strip**

First, the carboxylated MagMQD@Si-COOH was obtained by mixing ethanol solution of TEPSA and ultrasonic treatment. Then, the antibody-modified MagMQD@Si probe (immuno-MagMQD@Si) was prepared by directly coupling antibacterial antibody to the surface of MagMQD@Si-COOH. In brief, 1 mL of prepared MagMQD@Si-COOH was magnetically collected to remove ethanol, resuspended in 0.5 mL of 10 mM MES solution (pH 6.0), then reacted with 5 μL of freshly prepared 0.1 mM EDC and 5 μL of 0.2 mM NHS. After 15 min of incubation, activated MagMQD@Si-COOH was separated by magnetic enrichment, redispersed in 200 μL of 10 mM PBS solution (pH 7.4), and incubated with 6 μg of anti-*S. typhi* antibody for 2 h. Next, 80 μL of BSA (10% w/v) was added into the mixture to block any unreacted sites of MagMQD@Si-COOH. This process lasted for 30 min. The resulting immuno-MagMQD@Si probes were collected, redispersed in 0.5 mL of PBS solution (pH 7.4), then processed with vacuum freeze drying into a dry powder. Finally, 1 mg of immuno-MagMQD@Si was weighed and redispersed in 0.5 mL of preservation buffer (10 mM PBS containing 1% BSA [w/v], 0.05% Tween 20 [w/v], and 0.02% NaN_3_ [w/v]) for ICA detection. The ICA strips for immuno-MagMQD@Si probe using were consistent with antibody-conjugated MagMQD@Si-based ICA strip.

**S1.4 Preparation of AuNP-based ICA strip**

The AuNP-based ICA strip was prepared according to previously reported literature. First, AuNPs (20 nm) were fabricated through the citrate reduction method. Briefly, 200 mL of HAuCl_4_ solution (0.01%, w/v) was heated to the boiling point with stirring. Then, 2.2 mL of trisodium citrate (1%, w/v) was added rapidly to the boiling solution. The suspension was boiled for 15 min and then allowed to reach thermal equilibrium at room temperature, which yielded the Au NPs with a diameter of ~20 nm. Afterwards, the pH of 4 μg anti–bacteria antibody was adjusted to 9 with 0.2 M K_2_CO_3_ and incubated with 1 mL 20 nm AuNP (pH 8–9) for 60 min. Then, 50 μL of 10% BSA was added to block the unreacted sites of AuNPs. The as–prepared immuno–AuNPs were collected by centrifugation (5700 rpm, 6 min), and resuspended with 200µL of storage solution (10 mM PB solution containing 1% BSA (w/v), 0.1% PVP (w/v), 10% sucrose (w/v), and 0.05% Tween–20 (v/v)). Finally, it was dispensed onto the glass fiber paper and dried to prepare a conjugate pad. Then, the conjugate pad was assembled on the LFIA strip and cut it into a 3.0 mm strip for subsequent use.

**S1.5 Preparation of 200 nm Fe_3_O_4_ nanoparticles**

The 200 nm Fe_3_O_4_ NPs were synthesized by using a modified solvothermal reaction. Briefly, FeCl_3_·6H_2_O (2 mmol, 540 mg) was dissolved in a mixture of EG and DEG (V_EG_/V_DEG_ = 15/5; total volume of 40 mL) under magnetic stirring for 30 min. Subsequently, 2 g of PVP was added to the above solution, which was heated at 120 °C for 20 min until a transparent solution was obtained. Then, heating was stopped, and 4.5 g of NaOAc was added into the above solution. After vigorous stirring for an additional 30 min, the mixture was transferred into a Teflon-lined autoclave (50 mL capacity) and heated at 200 °C for 10 h. The obtained products were washed thrice with ethanol and water before they were dried under vacuum at 60 °C for 5 h and stored until future use. Finally, 5 mg of Fe_3_O_4_ MNPs was weighed and dissolved in 1 mL of deionized water for further use.

**S1.6 Preparation of bacterial samples with fixed concentration**

The concentrations of the used pathogens (*P. aeruginosa*, *S. pneumoniae*, and *S. typhi*) were determined by the conventional plate counting method. Briefly, the bacterial strain was inoculated into 5% sheep blood agar plates at 37 °C in an incubator containing 5% CO_2_ overnight. Several colonies were directly isolated from the plates and transferred into 1 mL of [sterile](javascript:;) PBS solution (10 mm, pH 7.4). The as-prepared bacterial solution was diluted 1×10^5^–1×10^8^ times with sterile water, and 0.1 mL of the diluted sample was coated to blood agar plates for 12 h. The colony forming units (CFU) on the plates were counted to determine the concentration of the original bacteria samples. Finally, the *P. aeruginosa*, *S. pneumoniae*, and *S. typhi* samples with defined concentration (10^6^–10 cells/mL) were prepared by dilution of the original bacteria solution.


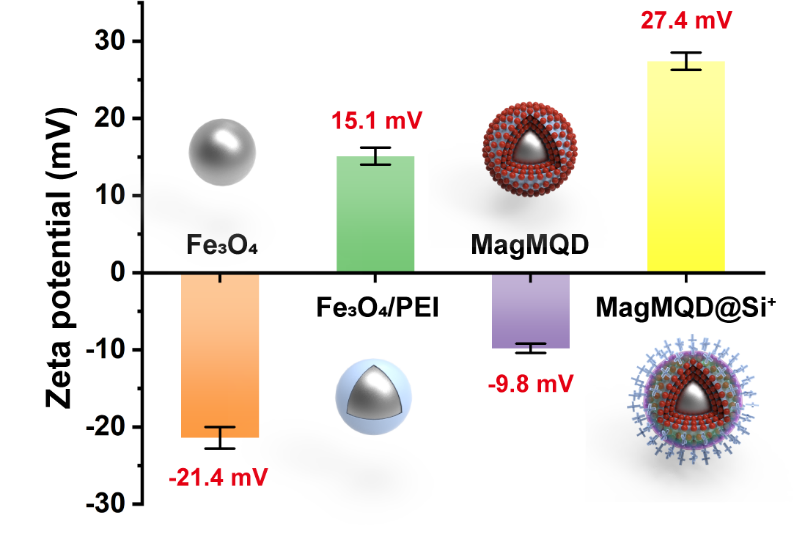


**Fig. S1** The change of Zeta potential during the synthesis of MagMQD@Si^+^. The error bar represents the standard deviation calculated from the five sets of samples (*n*=5).


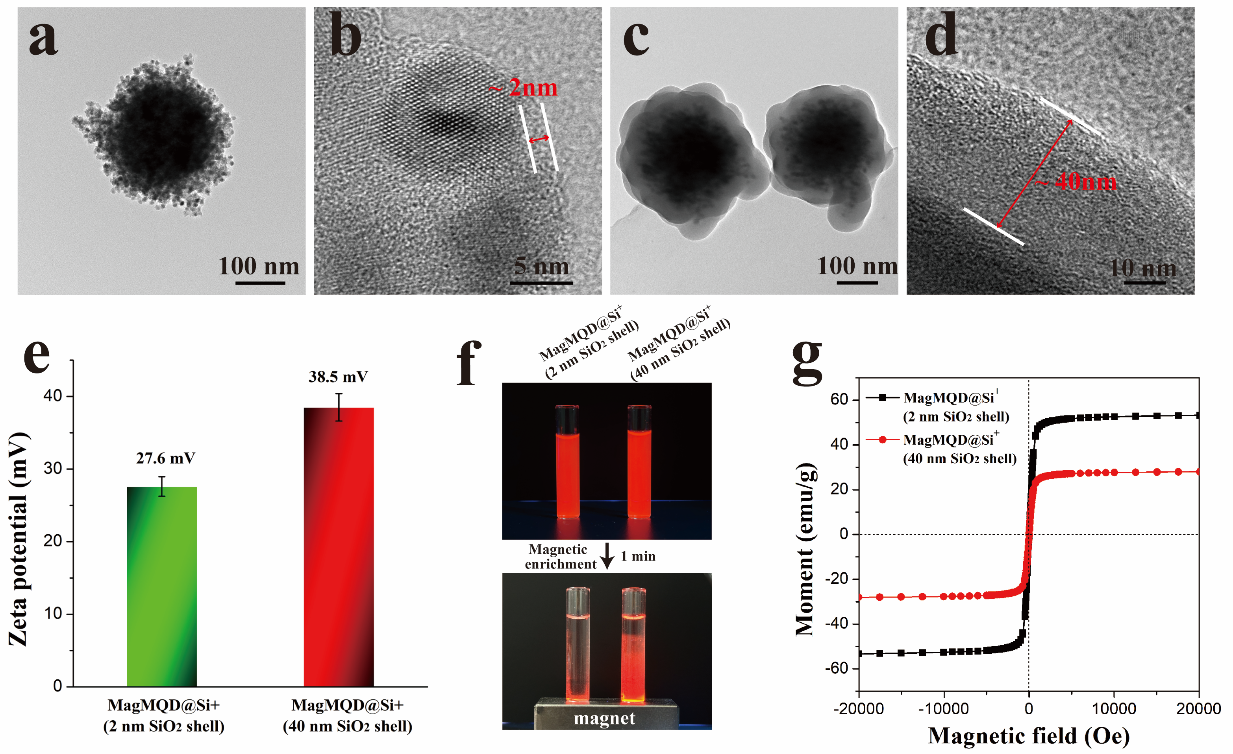


**Fig. S2** TEM image (a) and local amplification HRTEM image (b) of MagMQD@Si^+^ (2 nm SiO_2_ shell). TEM image (c) and local amplification HRTEM image (d) of MagMQD@Si^+^ (40 nm SiO_2_ shell). (e) Zeta potential of MagMQD@Si^+^ (2 nm SiO_2_ shell) and MagMQD@Si^+^ (40 nm SiO_2_ shell). (f) Magnetic separation and enrichment ability of MagMQD@Si^+^ (2 nm SiO_2_ shell) and MagMQD@Si^+^ (40 nm SiO_2_ shell). (g) Magnetic hysteresis curves of MagMQD@Si^+^ (2 nm SiO_2_ shell) and MagMQD@Si^+^ (40 nm SiO_2_ shell).


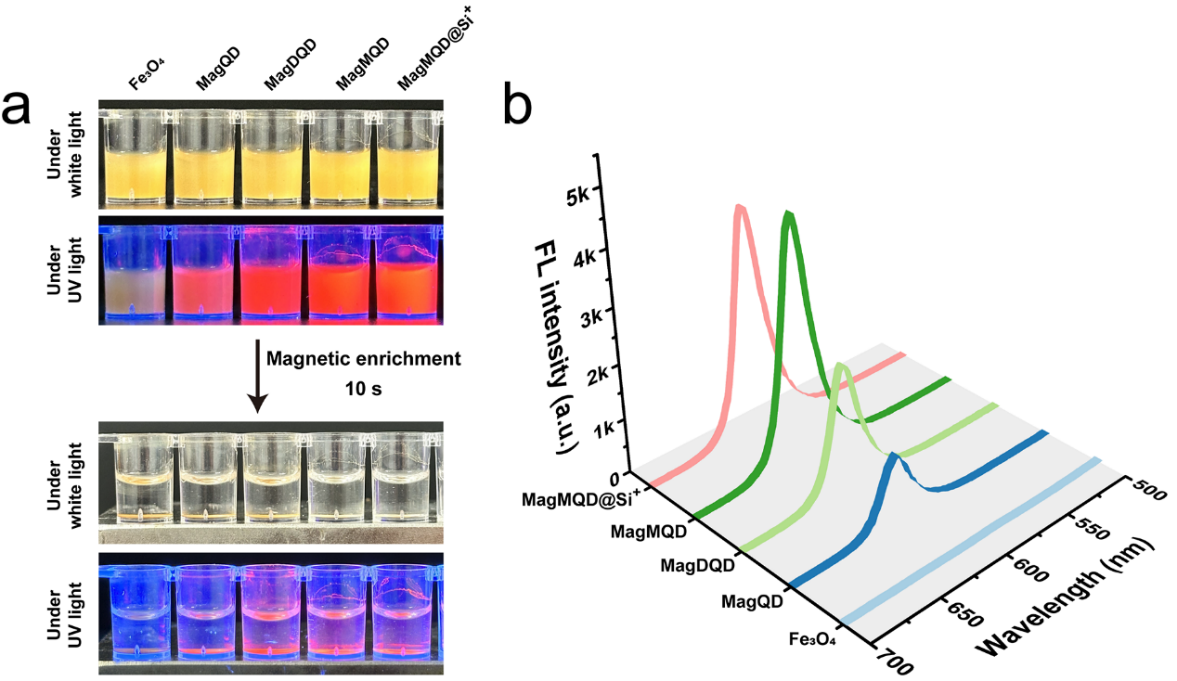


**Fig. S3** Comparison of magnetic enrichment ability (a) and fluorescence intensity (b) of Fe_3_O_4_, MagQD, MagDQD, MagMQD, MagMQD@Si^+^.

**S2 Calculation of maximum number of QDs on the Mag****MQD**

Here, we estimated the maximum average number of CdSe/ZnS QDs on the surface of MagMQD by using the following model.

**Model description**

In our model, there is a big sphere $O_{0}$, whose radius is denoted as $r_{1}$. And there are some small balls, whose radius is denoted as $r_{2},$attached to the surface of the big sphere. We want to know how many the small balls can completely cover the surface of the big sphere.

On the surface of the sphere $O_{0}$, the small balls are displayed as Fig. S4a. Three balls are tangent to three points on the surface of the sphere. And the three centers of the balls can be connected into an equilateral triangle, the edge length of which is $2r_{2}$. As Fig. S4b shows, if the sphere is fully covered by the small balls, it will be wrapped up by the spheroid composed of many equilateral triangles. Here, we make an assumption that the small balls can hold together on the surface of the sphere and form a buckyball.^[1]^ One property of the buckyball is that it can be formed by 12 regular pentagons and some regular hexagons. let $f$ denote the number of these pentagons and hexagons, $e$ denote the number of edges and $v$ denote the number of vertices in the buckyball. Then there is another property expressed by the following equation:

$f-e+v=2$.

Let $n_{0}$, $n_{1}$ separately denote all the number of vertexes and triangles in Fig. S4b. Here, $n_{0}$ also denotes the number of the small balls, which is what we want to estimate. Then the relationship between $n_{0}$ and $n_{1}$ can be inferred as the following equation:

$n_{1}=2n_{0}-4$.

Next, we use the area of two spheres to estimate the area of the spheroid in Fig. S4b. For the two spheres, one is the minimum circumscribed sphere of the spheroid, the other is the maximum inscribed sphere of the spheroid. The radiuses of two spheres are $r_{1}+r_{2}$ and $\sqrt{r_{1}^{2}+{2r}_{1}r_{2}-\frac{1}{3}r_{2}^{2}}$, which separately refer to $O_{0}O_{1}$ and $O_{0}O_{4}$ in Fig. S3c. Let $S_{2}$ and $S_{3}$ separately denote the area of the two spheres. And the area of each triangle in Fig. S4b is denoted as $S_{1}$. Then the following inequality will be satisfied:

$${S_{3}<n_{1}S_{1}<S}_{2}$$

After a series of calculations, we can get the estimate for $n_{0}$:

$$\frac{2\sqrt{3}\pi(r_{1}^{2}+{2r}_{1}r_{2}-\frac{1}{3}r_{2}^{2})}{3r_{2}^{2}}+2<n_{0}<\frac{2\sqrt{3}\pi{(r_{1}+r_{2})}^{2}}{3r_{2}^{2}}+2$$

In our study, for MagQD, 200 nm Fe_3_O_4_ core is the big sphere, and 12 nm CdSe/ZnS QDs were the small balls, thus $r_{1}$= 100 and $r_{2}$= 6;

For MagDQD, 212 nm MagQD is the big sphere, and 12 nm CdSe/ZnS QDs were the small balls, thus $r_{1}$= 106 and $r_{2}$= 6;

For MagMQD, 224 nm MagDQD is the big sphere, and 12 nm CdSe/ZnS QDs were the small balls, thus $r_{1}$= 112 and $r_{2}$= 6;

According to the formula above, the maximum average numbers of QDs on the first layer QD-shell, second layer QD-shell and third layer QD-shell can be figured out to 1129 < n < 1134, 1261 < n < 1266, and 1400 < n < 1405, respectively.

Thus, the maximum number of QDs onto single MagMQD with triple QD-shell can be estimated as about 3805 (1134+1266+1405).


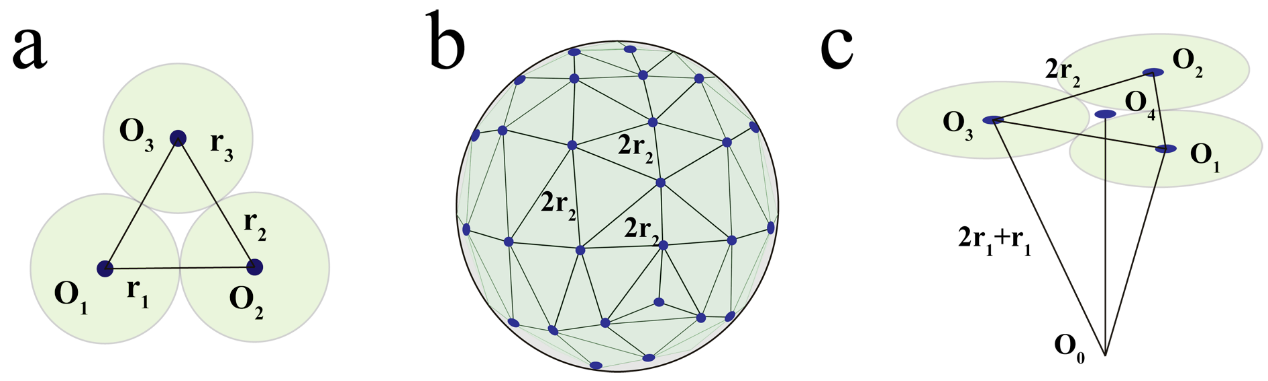


**Fig. S4** Distribution of small balls on the surface of the big sphere. (a) The location relationship of three small balls on the surface of the big sphere. They are tangent to three points. (b) Buckyball model composed of these smaller balls. Here, each vertex stands for a ball (center of the ball). (c) Description of the radius of two spheres used for estimating the area of the spheroid in (b). $O_{0}O_{1}$ denotes the distance of two centers of the bigger sphere and the smaller ball. The sphere with radius $O_{0}O_{1}$ is the minimum circumscribed sphere of the spheroid. $O_{0}O_{4}$ denotes the distance between the center of the big sphere and the triangle composed of three centers of the small balls. $O_{0}O_{4}\perp\triangle O_{1}O_{2}O_{3}$. The sphere with radius $O_{0}O_{4}$ is the maximum inscribed sphere of the spheroid.

**References**

1. Chung, F.; Sternberg, S., Mathematics and the Buckyball. *American Scientist* **1993,** 81 (1), 56-71.


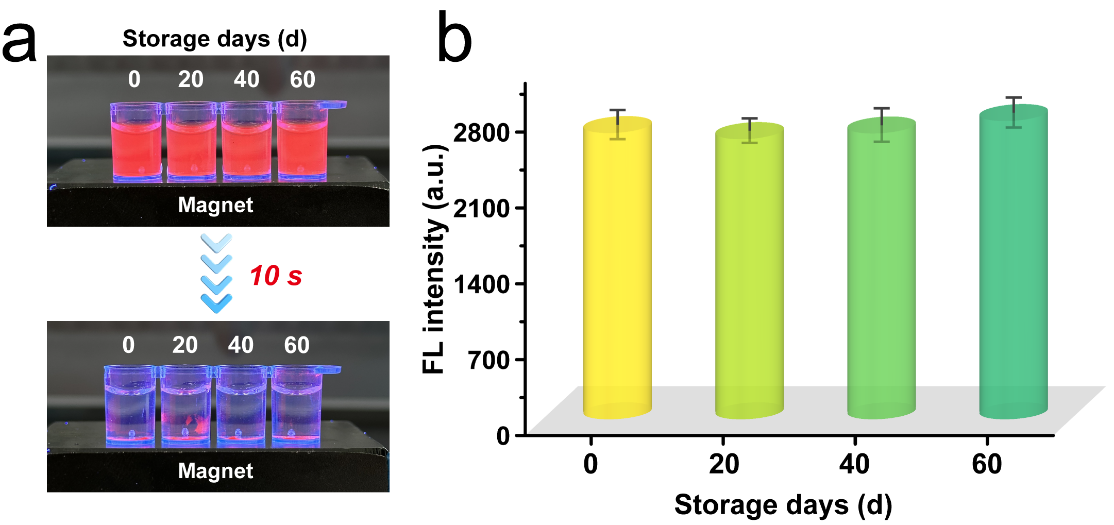


**Fig. S5** (a) Photographs and (b) fluorescence intensity of MagMQD@Si^+^ probe at different storage times. The error bar represents the standard deviation calculated from the five sets of samples (*n*=5).


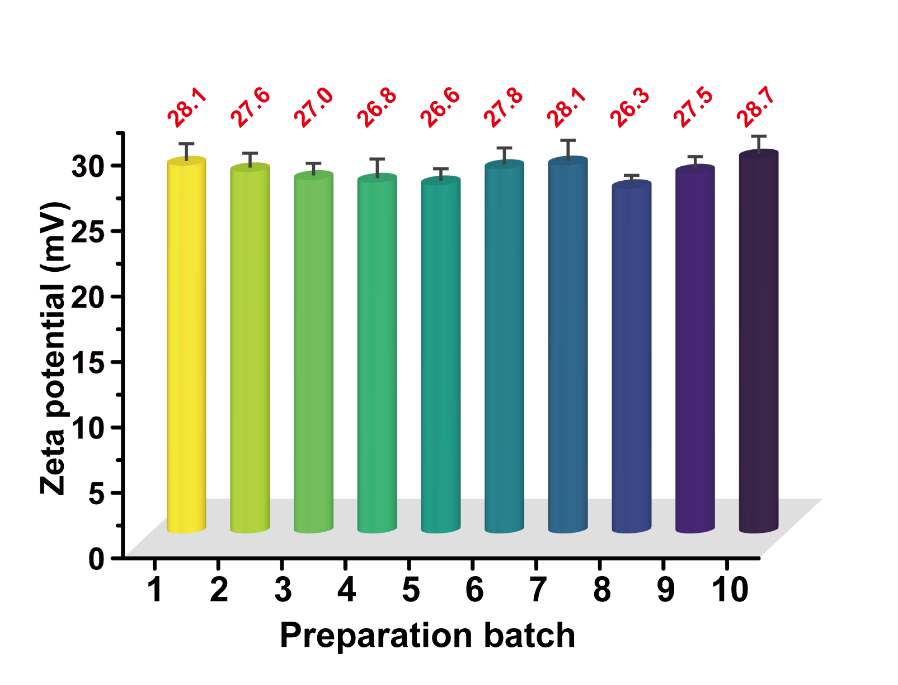


**Fig. S6** Zeta potential values of 10 batches of MagMQD@Si^+^ probes. The error bar represents the standard deviation calculated from the five sets of samples (*n*=5).

**S3.** [**Optimization**](javascript:;) **of electropositive probe-based ICA system**

To further improve the performance of MagMQD@Si^+^-based fluorescent ICA system, we then optimized key parameters including the antibody coating concentrations on the T lines, MagMQD@Si^+^ tag dosage, incubation time for bacterial capture, chromatographic time and running buffer composition. As shown in Fig. S7, when the concentrations of anti-*S. typhi* (a), anti- *S. pneumoniae* (b) and anti-*P. aeruginosa* (c) on the three T lines reached 1.2, 0.8 and 1.5 mg/mL, respectively, the established ICA achieved the highest SNR for pathogens detection. Fig. S8 and Fig. S9a showed that 6 µL of universal MagMQD@Si^+^ tags and 5 min of incubation times used for bacteria capture can create the highest SNR values of the fluorescence intensity on the T line of test strip. Fig. S9b showed that 10 min of chromatographic time is enough for MagMQD@Si^+^-based fluorescent ICA for different bacteria detection. Fig. S10 reveals that a PBS-based running buffer containing 1% Tween 20, 1% BSA, and 1% Milk generated the highest SNR on T lines.


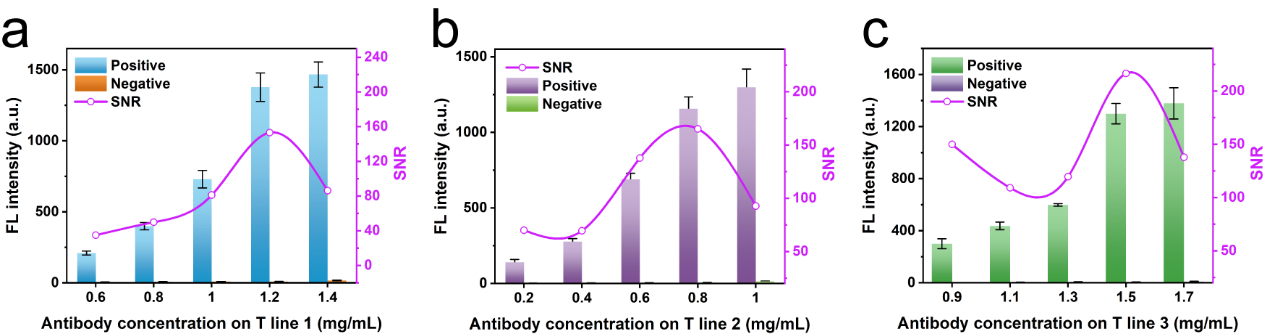


**Fig. S7** Optimization of anti-*S. typhi* (a), anti- *S. pneumoniae* (b) and anti-*P. aeruginosa* (c) concentration on the T line. The error bars indicate standard deviations calculated from three measurements. The error bar represents the standard deviation calculated from the five sets of samples (*n*=5).


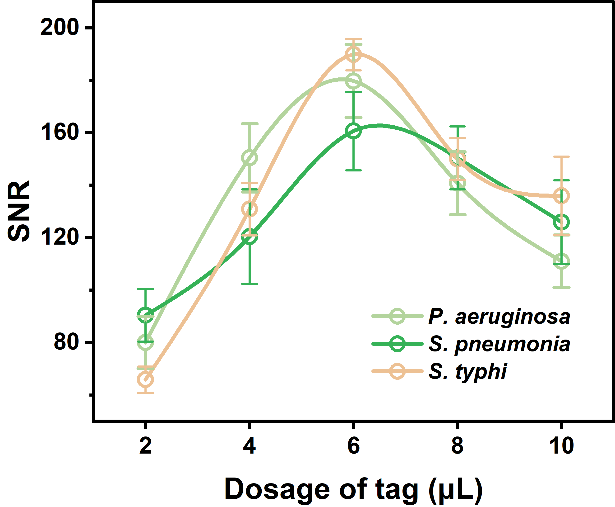


**Fig. S8** Optimization of tag amount of the MagMQD@Si^+^-based ICA system. The error bar represents the standard deviation calculated from the five sets of samples (*n*=5).


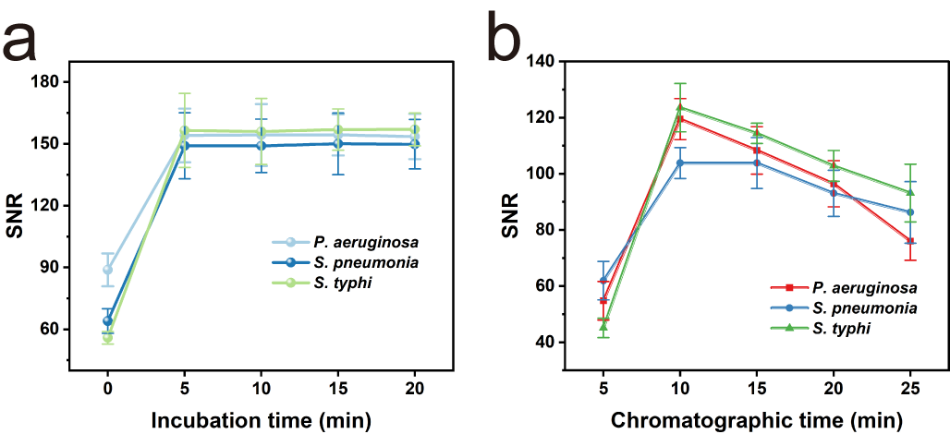


**Fig. S9** Optimization of incubation time (a) and chromatographic time (b) of the MagMQD@Si^+^-based ICA system. The error bar represents the standard deviation calculated from the five sets of samples (*n*=5).


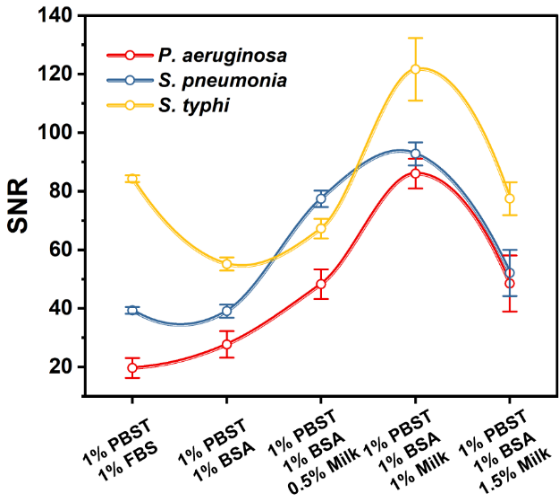


**Fig. S10** Optimization of running buffer composition of the MagMQD@Si^+^-based ICA system. The error bar represents the standard deviation calculated from the five sets of samples (*n*=5).


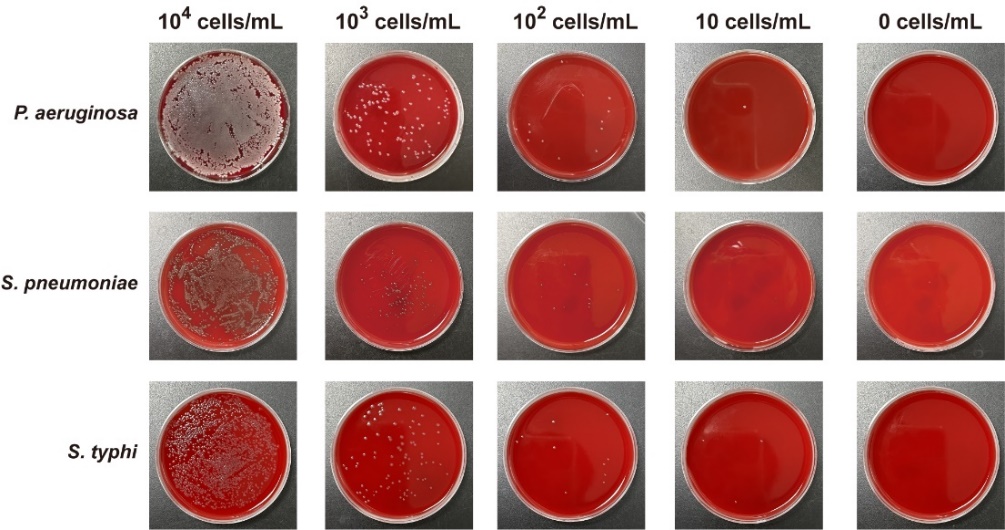


**Fig. S11** Plate counting method for the quantitative detection of *P. aeruginosa*, *S. pneumoniae*, and *S. typhi.*


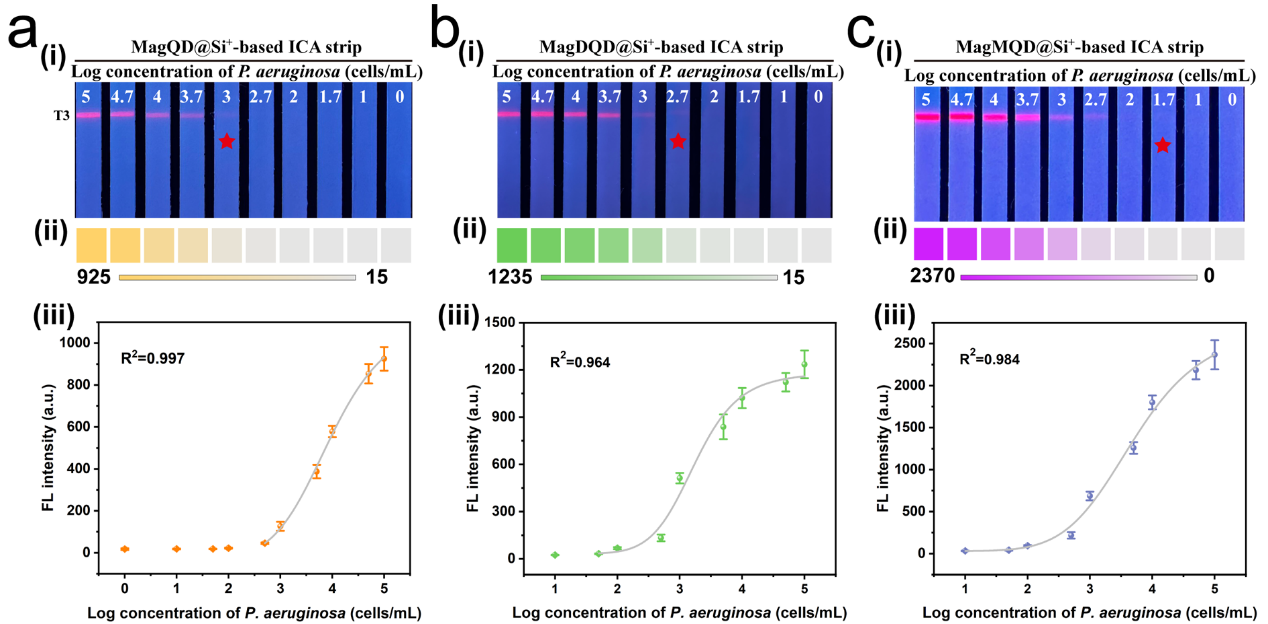


**Fig. S12** (i) Images, (ii) detailed fluorescence signal of the T line, and (iii) corresponding calibration curve of MagQD@Si^+^-based ICA (a), MagDQD@Si^+^-based ICA (b), and MagMQD@Si^+^-based ICA (c) for *P. aeruginosa.* The error bar represents the standard deviation calculated from the five sets of samples (*n*=5).

We compared MagMQD@Si^+^-based ICA and the ICA methods based on other magnetic QDs (MagQD@Si^+^ and MagDQD@Si^+^) to verify the effect of the multilayer QDs of the electropositive tag on fluorescence signal amplification. Fig. S12(i) and (ii) present the fluorescence images and measured fluorescence intensities of the tested strips in the MagQD@Si^+^-, MagDQD@Si^+^-, and MagMQD@Si^+^-ICA strips in the detection of the same concentrations of *P. aeruginosa* (10^5^–10 cells/mL). Obviously, the MagMQD@Si^+^ probe can generate higher fluorescence signals on the test line of ICA than the MagQD@Si^+^and MagDQD@Si^+^ probes. The red fluorescence signals on the T lines of the MagQD@Si^+^-, MagDQD@Si^+^-, and MagMQD@Si^+^-ICA strips could be observed with the naked eye at the concentrations of 10^3^, 500, and 50 cells/mL, respectively [Fig. S12a-c(i)]. The LODs of the MagQD@Si^+^-, MagDQD@Si^+^-, and MagMQD@Si^+^-based ICA methods were calculated to be 396, 77, and 9 cells/mL, respectively [Fig. S12a-c(iii)]. These results confirmed that using MagMQD@Si^+^ with multiple QD layers can effectively improve the detection sensitivity of ICA platform.


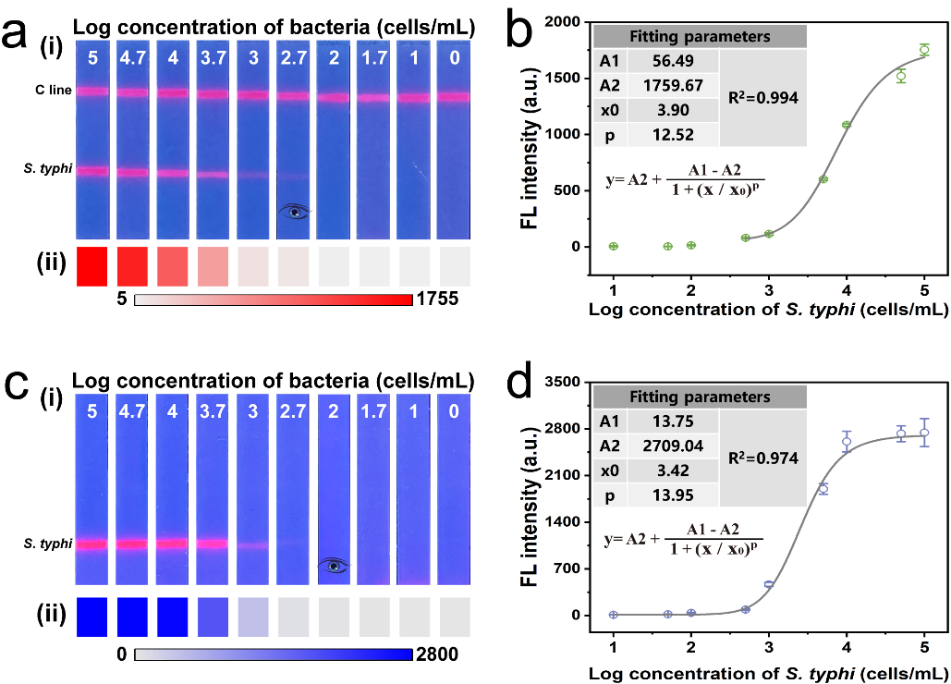


**Fig. S13** (a) Photographs (i) and corresponding fluorescence signals (ii) on the T lines and fitted curve (b) of antibody-conjugated MagMQD@Si-based ICA for *S. typhi* detection. (c) Photographs (i) and corresponding fluorescence signals (ii) on the T lines and fitted curve (d) of MagMQD@Si^+^-based ICA for *S. typhi* detection. The error bar represents the standard deviation calculated from the five sets of samples (*n*=5).


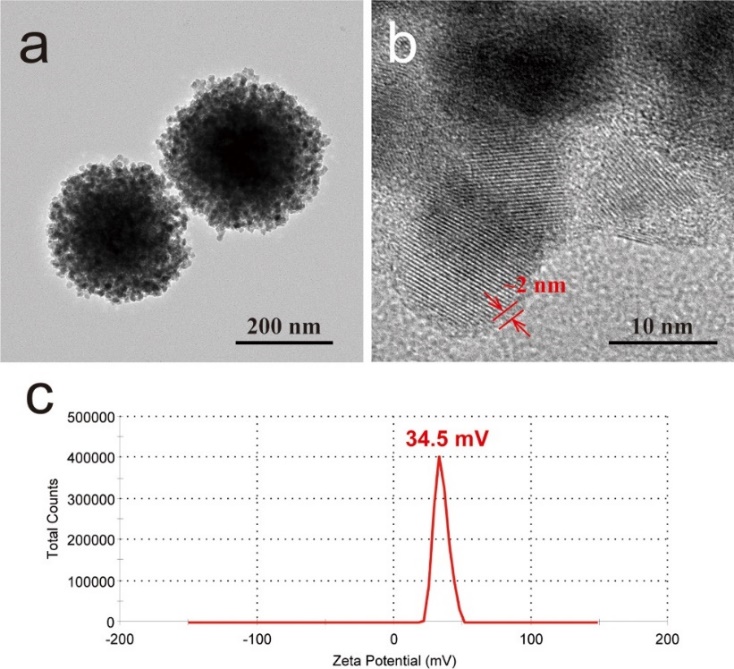


**Fig. S14** Characterization of the PEI-modified MagMQD probe (MagMQD^+^). (a) TEM image of MagMQD^+^ and (b) corresponding HRTEM image of the outer shell of MagMQD^+^ probe. (c) Zeta potential of MagMQD^+^ probe.

**Table S1.** Comparison of recently reported label-free ICA methods for bacteria detection.

| **Probe** | **Readout** | **Target** | **LOD**  **(cells/mL)** | **Assay time (min)** | | **Reference** |
| --- | --- | --- | --- | --- | --- | --- |
| Fe_3_O_4_@PDA@Pt | Colorimetric/ nano-enzyme | *E. coli* O157:H7 | 10-10^2^ | 35 | [1] | |
| Fe_3_O_4_@CuS | Photothermal/  Colorimetric | *E. coli* O157:H7 | 10^2^-10^3^ | 17 | [2] | |
| AIE probe | Fluorescence | *E. coli* O157:H7 | 10^5^ | 10 | [3] | |
| SIA-AuNPs | Colorimetric | Salmonella | 10^3^ | 14 | [4] | |
| FITC | Fluorescence | *E. coli* O157:H7 | 10^5^ | 5 | [5] | |
| SapYZUM13@  Mn_3_O_4_-NH_2_ | Colorimetric | *S. aureus* | 20 | 20 | [6] | |
| MnO_2_ nanosheet | Colorimetric | *S. enteritidis* | 10^3^ | 26 | [7] | |
| MagMQD@Si^+^ | Fluorescence | *P. aeruginosa*  *S. pneumoniae*  *S. typhi* | 8,  31,  40 | 15 | This  Research | |

**References**

[1] L. Dou, Y. Bai, M. Liu, S. Shao, H. Yang, X. Yu, K. Wen, Z. Wang, J. Shen, W. Yu, *Biosens. Bioelectron.* **2022**, *204*.

[2] M. Zhang, T. Bu, Y. Tian, X. Sun, Q. Wang, Y. Liu, F. Bai, S. Zhao, L. Wang, *Food Chem.* **2020**, *332*.

[3] C. Liu, S. Fang, Y. Tian, Y. Wu, M. Wu, Z. Wang, D. Xu, D. Hou, Q. Liu, *SLAS Technol.* **2021**, *26* (4), 377.

[4] Y. Ren, J. Wei, Y. He, Y. Wang, M. Bai, C. Zhang, L. Luo, J. Wang, Y. Wang, *Food Chem.* **2021**, *343*, 128518.

[5] C. Song, J. Li, J. Liu, Q. Liu, *Talanta.* **2016**, *156-157*, 42.

[6] Y. Han, W. Zhou, Y. Wu, A. Deng, L. Yuan, Y. Gao, H. Li, Z. Wang, B. Wang, G. Zhu, Z. Yang, *Food Chem* **2024**, *457*, 140189.

[7] Z. Deng, D. Yang, Y. Chen, X. Liu, Q. Wu, X. Yin, J. Wang, D. Zhang, *Chem. Eng. J.* **2023**, *477*, 147057.


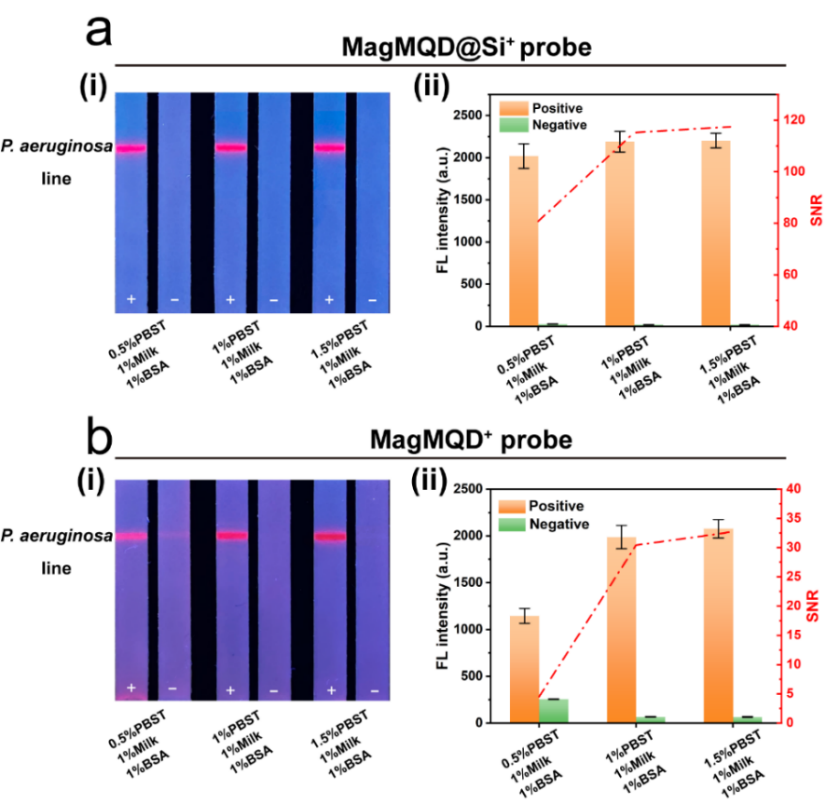


**Fig. S15** Comparison of the flow properties of (a) MagMQD@Si^+^ and (b) MagMQD^+^ probes on the test strips. The error bar represents the standard deviation calculated from the five sets of samples (*n*=5).


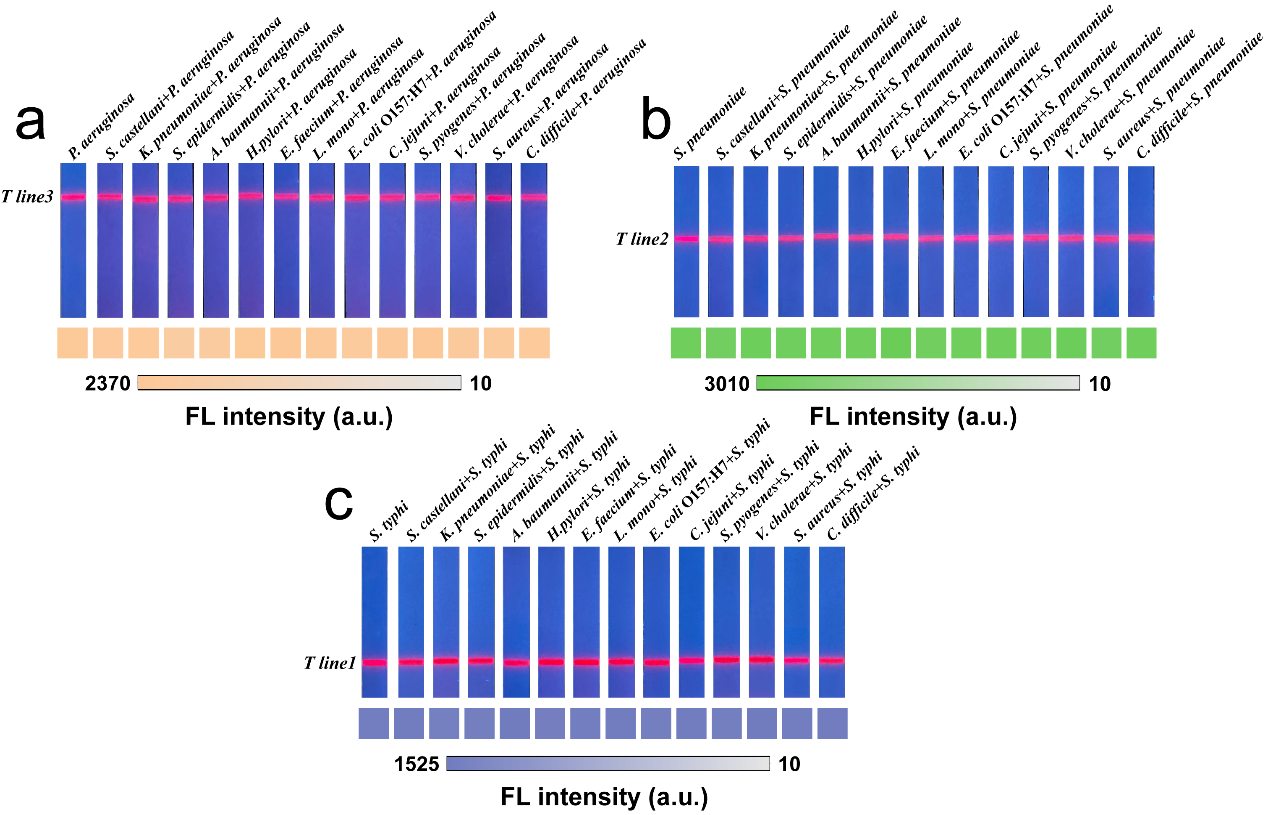


**Fig. S16** Selectivity testing of test strips in the presence of different non-target bacteria. Photographs of test strips and corresponding fluorescence intensity on (a) T3 line, (b) T2 line, and (c) T1 line for different bacteria samples.


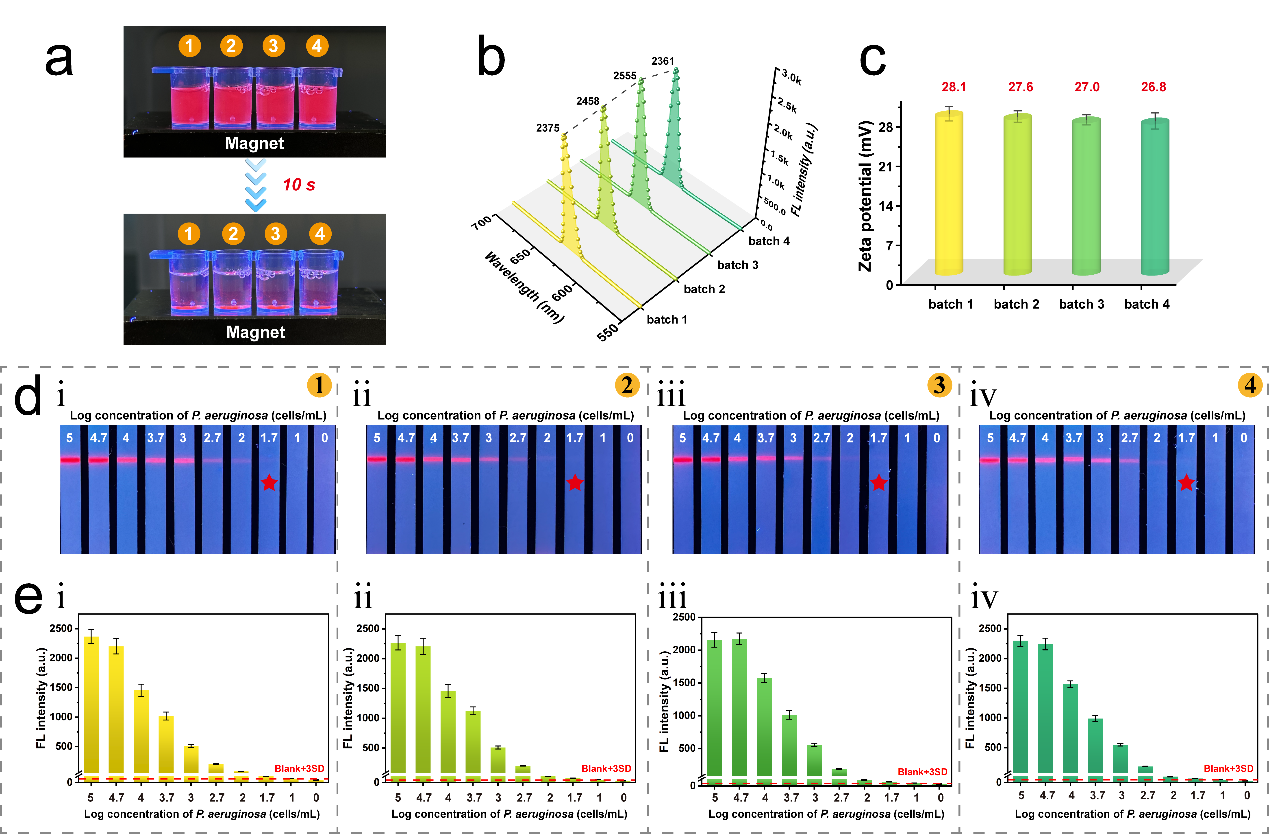


**Fig. S17** (a) Fluorescence image, (b) fluorescence emission spectra, and (c) zeta potential of four batches of MagMQD@Si^+^ probe stored for 2 months. (d-e) Detection results of MagMQD@Si^+^-based ICA method based on four batches of electropositive probe for *P. aeruginosa* at different concentrations (10^5^-10 cells/mL): (d) photographs of ICA strips and (e) corresponding fluorescence signal intensity on T lines. The error bar represents the standard deviation calculated from the five sets of samples (*n*=5).

As shown in Fig. S17a-b, the MagMQD@Si^+^ probes from different batches exhibited stable fluorescence intensity and their measured fluorescence signal reproducibility is rather good (RSD < 4.23%). The zeta potential results in Fig. S17c also demonstrated the four batches of MagMQD@Si^+^ probes have a stable surface electrical property after two months of storage. In addition, the reproducibility of MagMQD@Si^+^-based ICA method was assessed systematically by testing different batches of electropositive probe. As shown in Figs. S17d-e, all the tested ICA strips displayed good ﬂuorescence signal reproducibility on the test lines, and the measured ﬂuorescence signal with RSD values of less than 6.67%. These results confirmed the high reproducibility of proposed MagMQD@Si^+^ probe.


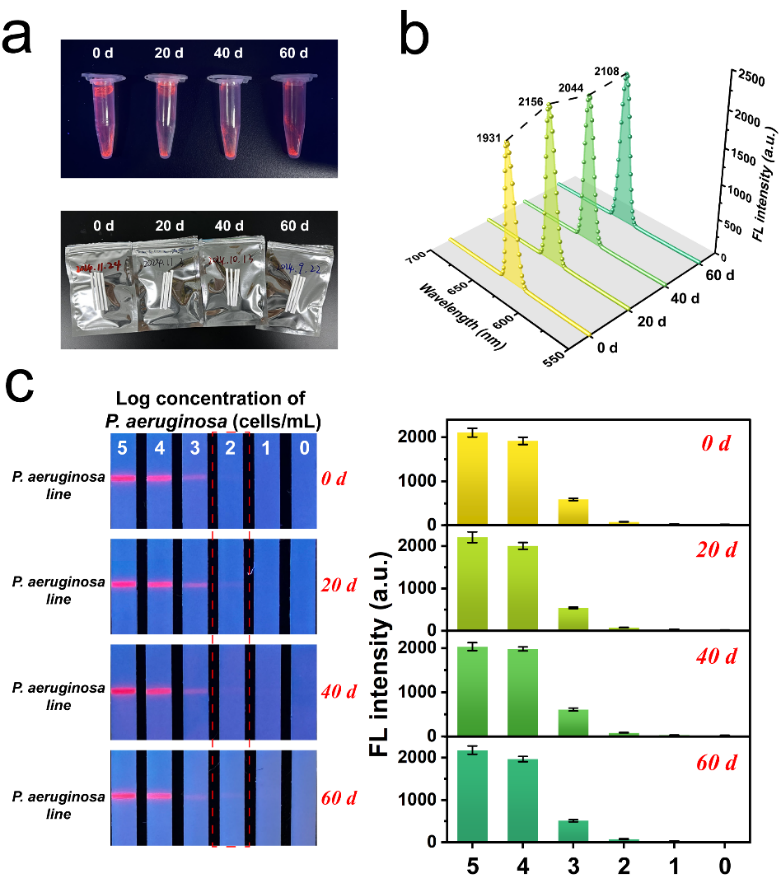


**Fig. S18** (a) Photographs of the MagMQD@Si^+^ probes and ICA strips and (b) fluorescence intensity of MagMQD@Si^+^ probes stored after different durations. (c) Assay stability of MagMQD@Si^+^−based ICA stored for different times (0-60 days). Error bars are the standard deviation of five repetitive tests. The error bar represents the standard deviation calculated from the five sets of samples (*n*=5).

The long-term storage conditions of MagMQD@Si^+^-ICA system has been studied systematically. Notably, the structure and components of the MagMQD@Si^+^-ICA strip are very simple. Only two main components including universal MagMQD@Si^+^ probe and antibody-modified test strip are used to build MagMQD@Si^+^-ICA, and these materials are easily preserved. For long-term preservation, the MagMQD@Si^+^ probes were divided into small pieces, prepared by vacuum freeze-drying and stored at a low temperature (4 °C) (Fig. S18a). The MagMQD@Si^+^ probes are stable for more than 60 days after the freeze-drying processing, and they can be restored to the original shape and form after being watered with the original biochemical peculiarity and show a stable fluorescence intensity (Fig. S18b). It is well known that ICA strip has good long-term stability, which is the basis of the numerous commercial colloidal gold test strips. Herein, the prepared ICA strips are well preserved in a sealed plastic bag containing desiccant gel at room temperature (Fig. S18a). We have detected the stability of MagMQD@Si^+^-ICA for bacteria detection as shown in Fig. S18c, and the fluorescence intensity of the test lines was rather stable after storage for 60 days. Thus, we can conclude the proposed MagMQD@Si^+^-ICA method has excellent long-term stability and potential for commercial application.

**Table S2.** The reproducibility of the MagMQD@Si^+^-based ICA for *P. aeruginosa*, *S. pneumoniae*, and *S. typhi* in spiked sample solutions (*n*=5).

| **Sample** | **Spiked**  **(cells/mL)** | **Detected (cells/mL)** | | | **Recovery (%)** | | | **RSD (%)** | | |
| --- | --- | --- | --- | --- | --- | --- | --- | --- | --- | --- |
|  |  | ***Pae*** | ***Spn*** | ***Sty*** | ***Pae*** | ***Spn*** | ***Sty*** | ***Pae*** | ***Spn*** | ***Sty*** |
| Lake | 5000 | 4541 | 4810 | 5232 | 90.82 | 96.19 | 104.64 | 9.43 | 3.87 | 8.88 |
|  | 500 | 494 | 493 | 530 | 98.83 | 98.50 | 105.96 | 1.99 | 2.37 | 3.03 |
|  | 50 | 48 | 50 | 52 | 95.53 | 100.08 | 102.99 | 9.48 | 4.40 | 2.47 |
| Throat swab | 5000 | 5106 | 4949 | 4941 | 102.12 | 98.98 | 98.83 | 7.22 | 7.44 | 11.41 |
|  | 500 | 483 | 515 | 482 | 96.60 | 103.00 | 96.38 | 2.97 | 9.67 | 9.97 |
|  | 50 | 54 | 47 | 49 | 107.69 | 93.05 | 97.22 | 5.60 | 6.13 | 6.02 |

**Note.** *Pae*, *Spn*, and *Sty* stands for *P. aeruginosa*, *S. pneumoniae* and *S. typhi*, respectively.

**S4. Detection of clinical samples**

**S4.1 Establishment of standard curve for detecting *P. aeruginosa* and *S. pneumoniae* by qPCR**

The prepared samples of *P. aeruginosa* and *S. pneumoniae* in different concentrations (10^8^–10 cells/mL) were tested by the lower respiratory tract bacterial nucleic acid detection Kit (Multiple fluorescent PCR) (Sansure Biotech Inc). The standard curve of qPCR method was then drawn according to the relationship between concentration and CT value.

**S4.2 Extraction of pathogenic nucleic acid from sputum**

Equal amount of 4% NaOH solution was added to the sputum sample, and after full mixing, it was liquefied at 37℃ for 30 min until there was no solid stringing. The bacteria in the liquefied sputum were centrifuged at 12000 rpm for 5 min and rinsed once with washing buffer (10 Mm Tris-HCl, 1 mM EDTA).

The precipitation of bacterial cells was suspended with 50 μL nucleic acid extraction solution, transferred to the nucleic acid extraction tube, and then heated at 95℃ for 5 min. At this time, the nucleic acid in the bacteria was released into the nucleic acid extraction solution. After heating, 12000 rpm, 5 min centrifuge, take the supernatant for use.

**S4.3 Detection of bacteria in clinical sputum samples by qPCR**

The 5 μL nucleic acid sample was added into the 45 μL PCR mixture and put into the nucleic acid amplification instrument. The HEX channel was used to detect *S. pneumoniae*, and the CY5 channel was used to detect *P. aeruginosa*. The following procedures are used for nucleic acid amplification:

**Table S3.** qPCR amplification procedure

| **Procedure** | | **Temperature** | **Time** | **Cycle number** |
| --- | --- | --- | --- | --- |
| 1 | UDG enzyme reaction | 50℃ | 2 min | 1 |
| 2 | Predegeneration | 94℃ | 3 min | 1 |
| 3 | Denaturation | 94℃ | 10 s | 45 |
| 4 | Anneal | 60℃ | 20 s |  |
| 5 | Extension and fluorescence detection | 75 ℃ | 20 s |  |
| 6 | Melting curve | 62–75 ℃ | Whole-process acquisition fluorescence | 1 |

**Detection result determination:**

If the HEX channel detects a typical S-shaped amplification curve with a Ct value < 39, the *S. pneumoniae* test is positive, and if Ct value > 39 or no Ct value is negative.

If the CY5 channel detects a typical S-shaped amplification curve and the Ct value < 39, the *P. aeruginosa* test result is positive; if the Ct value > 39 or no Ct value, it is negative.

**Table S4.** Quantification of 23 *P. aeruginosa* positive throat swab samples and 7 *S. pneumoniae* positive throat swab samples determined by the MagMQD@Si^+^-based ICA and qPCR.

| **Clinical**  **samples** | **MagMQD@Si^+^-based ICA** | | **qPCR** | |
| --- | --- | --- | --- | --- |
|  | **Classification** | **Quantification** | **Classification** | **Quantification** |
| 1 | *P. aeruginosa* | 8058 cells/mL | *P. aeruginosa* | 7244 cells/mL |
| 2 | *P. aeruginosa* | 901 cells/mL | *P. aeruginosa* | 1122 cells/mL |
| 3 | *P. aeruginosa* | 39172 cells/mL | *P. aeruginosa* | 42658 cells/mL |
| 4 | *P. aeruginosa* | 184332 cells/mL | *P. aeruginosa* | 165959 cells/mL |
| 5 | *P. aeruginosa* | 57628 cells/mL | *P. aeruginosa* | 61660 cells/mL |
| 6 | *P. aeruginosa* | 133110 cells/mL | *P. aeruginosa* | 114815 cells/mL |
| 7 | *P. aeruginosa* | 29911 cells/mL | *P. aeruginosa* | 31710 cells/mL |
| 8 | *P. aeruginosa* | 2615 cells/mL | *P. aeruginosa* | 2476 cells/mL |
| 9 | *P. aeruginosa* | 11636 cells/mL | *P. aeruginosa* | 12371 cells/mL |
| 10 | *P. aeruginosa* | 756 cells/mL | *P. aeruginosa* | 781 cells/mL |
| 11 | *P. aeruginosa* | 14889 cells/mL | *P. aeruginosa* | 13305 cells/mL |
| 12 | *P. aeruginosa* | 6628 cells/mL | *P. aeruginosa* | 6327 cells/mL |
| 13 | *P. aeruginosa* | 5038 cells/mL | *P. aeruginosa* | 6223 cells/mL |
| 14 | *P. aeruginosa* | 6446 cells/mL | *P. aeruginosa* | 5943 cells/mL |
| 15 | *P. aeruginosa* | 34550 cells/mL | *P. aeruginosa* | 32211 cells/mL |
| 16 | *P. aeruginosa* | 140751 cells/mL | *P. aeruginosa* | 99541 cells/mL |
| 17 | *P. aeruginosa* | 2151 cells/mL | *P. aeruginosa* | 2761 cells/mL |
| 18 | *P. aeruginosa* | 8885 cells/mL | *P. aeruginosa* | 8147 cells/mL |
| 19 | *P. aeruginosa* | 6298 cells/mL | *P. aeruginosa* | 7145 cells/mL |
| 20 | *P. aeruginosa* | 84778 cells/mL | *P. aeruginosa* | 90365 cells/mL |
| 21 | *P. aeruginosa* | 22108 cells/mL | *P. aeruginosa* | 27290 cells/mL |
| 22 | *P. aeruginosa* | 9934 cells/mL | *P. aeruginosa* | 11272 cells/mL |
| 23 | *P. aeruginosa* | 34711 cells/mL | *P. aeruginosa* | 39920 cells/mL |
| 24 | *S. pneumoniae* | 48320 cells/mL | *S. pneumoniae* | 50466 cells/mL |
| 25 | *S. pneumoniae* | 74977 cells/mL | *S. pneumoniae* | 67655 cells/mL |
| 26 | *S. pneumoniae* | 8518 cells/mL | *S. pneumoniae* | 7965 cells/mL |
| 27 | *S. pneumoniae* | 485 cells/mL | *S. pneumoniae* | 505 cells/mL |
| 28 | *S. pneumoniae* | 16884 cells/mL | *S. pneumoniae* | 12440 cells/mL |
| 29 | *S. pneumoniae* | 12210 cells/mL | *S. pneumoniae* | 15985 cells/mL |
| 30 | *S. pneumoniae* | 31336 cells/mL | *S. pneumoniae* | 33884 cells/mL |
